# Three New Asperentin Derivatives from the Algicolous Fungus *Aspergillus* sp. F00785

**DOI:** 10.3390/md12125993

**Published:** 2014-12-15

**Authors:** Qian Tang, Kai Guo, Xiao-Yang Li, Xiu-Ying Zheng, Xiang-Jian Kong, Zhong-Hui Zheng, Qing-Yan Xu, Xianming Deng

**Affiliations:** 1 State Key Laboratory of Cellular Stress Biology, School of Life Sciences, Xiamen University, Xiamen, Fujian 361102, China; 2 State-Province Joint Engineering Laboratory of Targeted Drugs from Natural Products, Xiamen University, Xiamen, Fujian 361102, China; E-Mails: 986713276@qq.com (Q.T.); guokai1129@qq.com (K.G.); xiaoyang19901107@sina.com (X.-Y.L.); zhzheng@xmu.edu.cn (Z.-H.Z.); 3 State Key Laboratory for Physical Chemistry of Solid Surface, College of Chemistry and Chemical Engineering, Xiamen University, Xiamen, Fujian 361005, China; E-Mails: zhengxiuying37@163.com (X.-Y.Z.); xjkong@xmu.edu.cn (X.-J.K.)

**Keywords:** asperentin, endophytic fungus, crop pathogen, α-d-ribofuranose

## Abstract

Three new asperentin-type compounds, 6-*O*-α-d-ribosylasperentin (**1**) and 6-*O*-α-d-ribosyl-8-*O*-methylasperentin (**2**) and 5-hydroxyl-6-*O*-methylasperentin (**3**), along with asperentin (**4**) and its known analogues (**5**–**9**), were isolated from a halotolerant *Aspergillus* sp. strain F00785, an endotrophic fungus from marine alga. Their structures were determined using extensive NMR and HRESIMS spectroscopic analysis, including the X-ray crystallographic data for the assignment of the absolute configurations of compound **9**. Compound **4** exhibited highly potent inhibitory activity against crop pathogens, *Colletotrichum gleosporioides* Penz. and *Colletotrichum gleosporioides* (Penz.) Sacc.

## 1. Introduction

**Figure 1:**
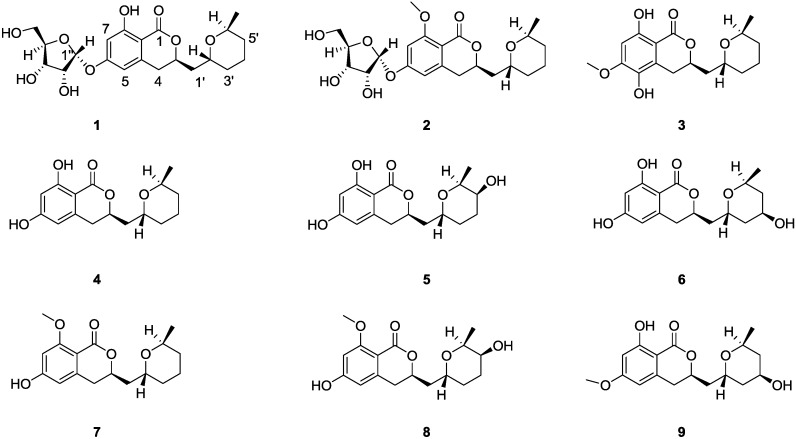
Structures of new asperentin analogs (**1**–**3**), (−)-asperentin (**4**) and its derivatives (**5**–**9**).

Marine microorganisms inhabiting relatively uninvestigated and extreme environments have been the focus of attractive sources for novel and bioactive secondary metabolites [1,2]. Marine natural products with broad spectra of bioactivities such as anti-tumor, anti-microtubule, photoprotective, antibiotic and anti-infective [3,4,5], are exceptionally interesting high value products for applications in the pharmaceutical industry. At the moment there are eight Food and Drug Administration (FDA) or European Medicines Agency (EMEA) approved drugs [6], for example trabectedin [7,8], an anti-tumor agent, and brentuximab vedotin [9], an antibody-drug conjugate. Moreover, several compounds are in different phases of the clinical trials [10,11]. Halotolerant microorganisms are microbes that are able to grow well in varied saline conditions, such as marine, salt lake, salt field, *etc.* [12,13]. It is believed that the high-salt environment might activate some silent genes and induce unique biosynthesis pathway in these microbes [14,15]. In the course of our ongoing search for marine-originated bioactive microbial metabolites, a halotolerant endogenic fungal *Aspergillus* sp. F00785 was isolated from the marine alga collected in Jinjiang Saltern, Fujian province, China. A solvent partition followed by repeated chromatographic purifications of the fermentation extracts afforded three new asperentin derivatives, 6-*O*-α-d-ribosylasperentin (**1**), 6-*O*-α-d-ribosyl-8-*O*-methylasperentin (**2**) and 5-hydroxyl-6-*O*-methylasperentin (**3**), along with (−)-asperentin (**4**), and five asperentin derivatives, 5′-hydroxyasperentin (**5**), 4′-hydroxyasperentin (**6**), asperentin-8-methyl ether (**7**), 5′-hydroxyasperentin-8-methyl ether (**8**) and 4′-hydroxyasperentin-6-methyl ether (**9**) (Figure 1). Here, we report the isolation, structure determination and biological activity of asperentin and its derivatives (**1**−**9**). The absolute configurations of stereocentres, C-2′, C-6′ and C-3 in **9**, were determined for the first time by X-ray crystallography. Compound **4** exhibited potent activity against *Colletotrichum gleosporioides* Penz. (*C. gleosporioides* Penz.) and *C. gleosporioides* (Penz.) Sacc.

## 2. Results and Discussion

### 2.1. Structure Elucidation

**Figure 2:**
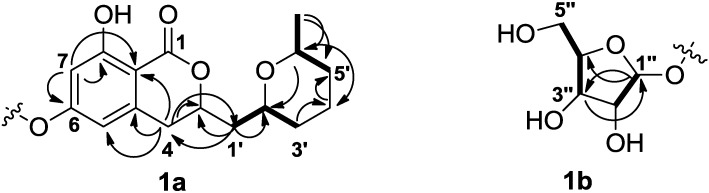
The structures of fragments **1a** and **1b** of 6-*O*-α-d-ribosylasperentin (**1**) and selected HMBCs (H**→**C) and ^1^H-^1^HCOSY correlations (bold line).

6-*O*-α-d-Ribosylasperentin (**1**) was isolated as pale yellow oil, which is soluble in acetone, chloroform and methanol. The R_f_ value was 0.3 on TLC over silica gel developed with CHCl_3_/MeOH (v/v, 10:1). The molecular formula of **1** was deduced as C_21_H_28_O_9_ based on the HRESIMS (*m/z* 447.1632 [M + Na]^+^, calculated for C_21_H_28_O_9_Na, 447.1631). The IR absorptions at 3364 and 1667 cm^−1^ suggested the presence of hydroxyl and carbonyl groups. The ^1^H- and ^13^C-NMR spectra of **1** in CDCl_3_ displayed signals for one methyl, six aliphatic methylenes, seven aliphatic methines, two *meta*-coupled aromatic methines, four aromatic quaternary carbons, and one ester carbonyl carbon (Table 1). The structure of fragment **1a** was established on the basis of HMBC correlations from H-4 to C-1′, C-3, C-8a, C-4a and C-5, H-7 to C-6, C-8 and C-8a, H-3′ to C-4′, and H-4′ to C-5′; and ^1^H-^1^H COSY correlations (H-1′/H-2′, H-2′/H-3′, H_3_-6′/H-6′, and H-6′/H-5′) (Figure 2). Its ^1^H and ^13^C NMR data are compatible with those reported for asperentin [16]. Fragment **1b** was further determined on the basis of HMBC correlations from H-3″ to C-1″, H-1″ to C-3″ and C-4″, and the ^1^H-^1^HCOSY correlations from H-5″ to H-4″, H-2″ to H-1″. Finally, fragments **1a** and **1b** were linked together via a glycosidic bond indicated by the H-1″−C-6 three-bond correlation in the HMBC spectrum.

Acid hydrolysis of **1** afforded a pentofuranose and (−)-asperentin (**4**) (${\left[\text{α}\right]}_{\text{D}}^{20}$ = −23°, *c* = 0.83, EtOH) [17]. The latter was also known as (−)-cladosporin [18], its absolute configuration of (*R*)-3,4-dihydro-3-(((2′*R*,6′*S*)tetrahydro-6′-methyl-2*H*-pyran-2-yl)-methyl)-6,8-dihydroxy-isochromen-1-one was confirmed by comparing its NMR and physiochemical data with literature data [17,18]. The pentofuranose was determined to be ribose on the basis of ^1^H- and ^13^C-NMR data [19]. The sugar moiety was determined as d-ribose by comparison of the optical rotation of its tetra-acetate derivative (${\left[\text{α}\right]}_{\text{D}}^{20}$ = −17°, *c* = 0.68, MeOH) with the reported data [20,21]. Additionally, the stereochemistry of the anomeric carbon of the d-ribofuranose moiety was determined as α-configuration on the basis of the chemical shift and coupling constant of C-1″ (δ_H_ 5.69 (d, *J* = 3.5 Hz), δ_C_ 100.1) that is consistent with the reported value [21].

The two hydrolysates of **1** further validated the structures of fragments **1a** and **1b**. With all the obtained data, the structure of 6-*O*-α-d-ribosylasperentin (**1**) was deduced as (*R*)-3,4-dihydro-3-(((2′*R*, 6′*S*) tetrahydro-6′-methyl-2*H*-pyran-2′-yl)-methyl)-6-*O*-(α-d-ribofuranosyl)-8-hydroxyl-isochromen-1-one, an asperentin-6-*O*-riboside.

6-*O*-α-d-Ribosyl-8-*O*-methylasperentin (**2**) was isolated as pale yellow oil, which is soluble in acetone, chloroform and methanol. The R_f_ value was 0.35 eluting with the mixed solvent of CHCl_3_/MeOH (v/v, 10:1). The molecular formula of **2** was deduced as C_22_H_30_O_9_ based on the HRESIMS (*m/z* 439.1975 [M + H]^+^, calculated for C_22_H_31_O_9_, 439.1968). Analysis of the IR spectrum indicated the presence of hydroxyl and carbonyl functionalities with IR absorption at 3445 and 1700 cm^−1^, respectively.

**Table 1:** ^1^H and ^13^CNMR data for **1**–**4** in CDCl_3_.

No.	1		2		3		4	
	δ_H_^a^ (*J* in Hz)	δ_C_^b^	δ_H_^a^ (*J* in Hz)	δ_C_^b^	δ_H_^a^ (*J* in Hz)	δ_C_^b^	δ_H_^a^ (*J* in Hz)	δ_C_^b^
1		169.6, C		161.6, C		170.0, C		169.9, C
3	4.72, m	76.5, CH	4.60, m	74.6, CH	4.63, m	76.3, CH	4.72, m	76.4, CH
4	2.87, m	33.6, CH_2_	2.92, dd, (16.2, 11.5)	35.4, CH_2_	3.10, dd, (16.8, 3.4)	27.3, CH_2_	2.86, m	33.7, CH_2_
			2.83, dd, (16.2, 2.9)		2.62, dd, (16.8, 11.5)			
4a		141.5, C		144.0, C		122.6, C		141.8, C
5	6.42, s	107.6, CH	6.56, d, (2.0)	106.8, CH		134.3, C	6.32, s	106.7, CH
OH-5					5.20, s			
6		162.5, C		162.6, C		153.1, C		162.9, C
OCH_3_-6					3.86, s	56.2, CH_3_		
7	6.56, s	102.9, CH	6.61, d, (2.0)	100.1, CH	6.34, s	97.8, CH	6.19, s	102.0, CH
8		163.8, C		162.9, C		157.8, C		164.3, C
OCH_3_-8			3.94, s	56.3, CH_3_				
8a		103.2, C		106.8, C		100.7, C		101.7, C
1′	1.98, m; 1.82, m	39.2, CH_2_	1.92, m; 1.83, m	39.5, CH_2_	1.95, m; 1.78, m	39.5, CH_2_	1.99, m; 1.87, m	39.4, CH_2_
2′	4.10, m	66.4, CH	4.10, m	66.2, CH	4.05, m	66.3, CH	4.13, m	66.5, CH
3′	1.69, m; 1.35, m	30.8, CH_2_	1.36, m	30.9, CH_2_	1.64, m; 1.29, m	30.8, CH_2_	1.73, m; 1.38, m	30.9, CH_2_
4′	1.70, m; 1.62, m	18.2, CH_2_	1.73, m; 1.64, m	18.3, CH_2_	1.65, m; 1.56, m	18.3, CH_2_	1.74, m; 1.65, m	18.2, CH_2_
5′	1.68, m; 1.32, m	31.3, CH_2_	1.72, m	31.0, CH_2_	1.63, m; 1.26, m	31.1, CH_2_	1.72, m; 1.36, m	31.0, CH_2_
6′	3.94, m	67.6, CH	3.96, m	67.7, CH	3.89, m	67.4, CH	4.02, m	67.9, CH
CH_3_-6′	1.21, d, (4.1)	19.1, CH_3_	1.22, d, (6.5)	18.9, CH_3_	1.14, d, (6.5)	19.2, CH_3_	1.25, d, (6.5)	18.9, CH_3_
1″	5.69, d, (3.5)	100.1, CH	5.77, d, (4.5)	100.5, CH				
2″	4.29, brs	72.1, CH	4.34, m	72.2, CH				
3″	4.19, brs	70.1, CH	4.24, dd, (6.2, 2.7)	70.4, CH				
4″	4.21, brs	86.4, CH	4.28, dd, (6.2, 2.7)	86.5, CH				
5″	3.78, d, (11.6); 3.74, d, (11.6)	62.3, CH_2_	3.89, dd, (12.1, 3.1); 3.81, dd, (12.1, 3.1)	62.5, CH_2_				
8-OH	11.13, s				10.86, s		11.10, s	

^a^ 600 MHz; ^b^ 150 MHz.

The structure of **2** was determined as 8-methoxyl analogue of **1** on the basis of the similar NMR data of both compounds with the exception of the absence of a hydroxyl group and the presence of a methoxyl at C-8 (δ_H-OMe_ 3.94, δ_c-OMe_56.3) (Table 1). That the methoxyl substituent on C-8 was further confirmed by HMBC correlation from OCH_3_ (δ_H_ 3.94) to C-8 (δ_C-8_ 162.9). Thus, **2** was 8-methoxyasperentin-6-*O*-riboside (Figure 1).

5-Hydroxyl-6-*O*-methylasperentin (**3**), another structurally similar metabolite, was isolated as white needle, which is soluble in acetone, chloroform and methanol. The molecular formula of **3** was deduced as C_17_H_22_O_6_ based on the HRESIMS (*m/z* 345.1308 [M + Na]^+^, calculated for C_17_H_22_O_6_Na, 345.1314). The IR absorptions at 3319 and 1657 cm^−1^ suggested the presence of hydroxyl and carbonyl groups. The NMR spectra were closely related to those of fragment **1a**, except that the signals (δ_H-5_ 6.42, δ_C-5_ 107.6) of **1a** was replaced with an aromatic oxygenated quaternary carbon (δ_c_ 134.3) which indicated a hydroxyl-substitution at C-5 (Table 1). Additionally, HMBC correlations from phenol hydrogen (δ_H_5.20) at C-5 to C-4a (δ_C-4a_ 122.6), C-5 (δ_C-5_ 134.3) and C-6 (δ_C-6_ 153.1), and from OCH_3_ (δ_H_ 3.86) to C-6 (δ_C-6_ 153.1) further confirmed that **3** was 5-hydroxyasperentin-6-methyl ether.

**Figure 3:**
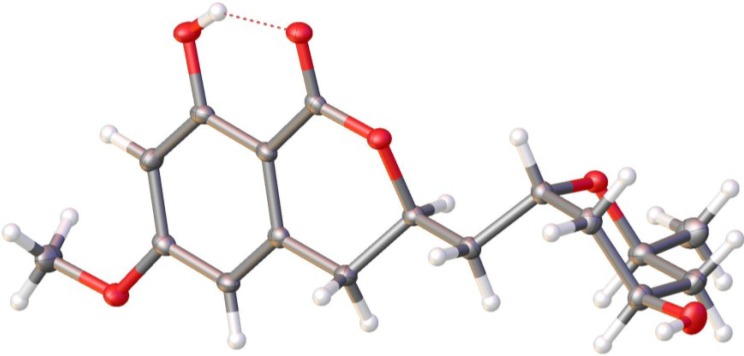
X-ray crystal structure of **9**.

Compounds **4**−**9** were isolated along with 6-*O*-α-d-ribosylasperentin (**1**), 6-*O*-α-d-ribosyl-8-*O*-methylasperentin (**2**) and 5-hydroxyl-6-*O*-methylasperentin (**3**). By analyses and comparisons of their NMR spectra, MS data and specific rotations with those reported in the literatures [17,18,22], compounds **4**−**9** were identified as (−)-asperentin (**4**) [17,18], 5′(*S*)-hydroxy-asperentin (**5**) [22], 4′(*R*)-hydroxyasperentin (**6**) [22], asperentin-8-methyl ether (**7**) [22], 5′(*S*)-hydroxy-asperentin-8-methyl ether (**8**) [22] and 4′(*R*)-hydroxy-asperentin-6-methyl ether (**9**) [22], respectively. The absolute configuration of stereocentres, C-2′, C-6′ and C-3 in **9**, was determined as (*R*)-8-hydroxy-3-(((2′*R*,4′*R*,6′*S*)-4-hydroxy-6-methyltetrahydro-2*H*-pyran-2-yl)methyl)-6-methoxy-isochroman-1-one by the results of X-ray single-crystal diffraction using Flack parameters (Figure 3 and please see Supplementary Information). An intramolecular hydrogen bond was observed, which is consistent with the NMR data of δ_8-OH_ (11.20), downfield shift compared with the free phenol hydrogen.

### 2.2. Bioactivity Results

The antifungal activity of compounds **1**–**9** against three crop pathogens, *C. gleosporioides* Penz, *C. gleosporioides* (Penz) Sacc. and *Botrytis cinerea* Pers, were evaluated by filter-paper disk method using amphotericin B as positive control. The results showed that only (−)-asperentin (**4**) exhibited strong inhibitory activity and no activity were observed for the other compounds. At a concentration of 5 mg/mL, the inhibition zone of **4** to *C. gleosporioides* Penz. was 19.7 ± 0.58 mm, while that of amphotericin B was 15.7 ± 1.25 mm (Table 2).

**Table 2:** Antimicrobial activity of (−) asperentin (**4**).

Compounds	Inhibition Zone (mm)		
	*C. gleosporioides* Penz	*C. gleosporioides* (Penz) Sacc.	*B. cinerea* Pers
**(−)-asperentin (4)**	19.7 ± 0.58	13.3 ± 3.40	1.67 ± 1.11
**amphotericin B**	15.7 ± 1.25	16.0 ±1.41	11.0 ± 0.82

## 3. Experimental Section

### 3.1. General Experimental Procedures

Optical rotations were measured using a Perkin-Elmer 341 polarimeter (PerkinElmer Inc., Waltham, MA, USA). UV spectra were recorded on Jasco V-530 spectrophotometer (JASCO International Co., Tokyo, Japan). IR spectra were obtained on Perkin-Elmer 552 spectrophotometer. NMR spectra were recorded on a Bruker Avance-600 spectrometer (600 MHz) (Bruker Co., Bremen, Germany) using TMS as the internal standard. ESI-MS was measured on a Thermo-Finnigan LCQ Advantage mass spectrometer (Thermo Fisher Scientific Inc, San Jose, CA, USA). HR-ESI-MS was obtained on a Bruker LC-QTOF mass spectrometer. Semi-preparative high pressure liquid chromatography (HPLC) was performed on Agilent 1200 using XDB C18 column (10 × 250 mm, 5 μm, flow = 2 mL/min) (Agilent Technologies Inc., Santa Clara, CA, USA). TLC detection was carried out using precoated silica gel GF_254_ plates (10–40 μm, Qingdao Marine Chemical Plant, Qingdao, China). Column chromatography was performed with silica gel (200–300 mesh, Qingdao Marine Chemical Plant, Qingdao, China), reverse phase RP-18 (40–63 μm, Merck, Darmstadt, Germany), and Sephadex LH-20 (Amersham Biosciences, Sweden). All solvents were of analytical grade.

### 3.2. Fungi Materials

The marine-derived endophytic fungus *Aspergillums* sp. strain F00785 was identified by morphological characteristics. It was isolated from marine alga, *Enteromorpha prolifera*, collected in Jinjiang Saltern, Fujian province, China, in 2006, and preserved in liquid paraffin at 4 °C until its use for fermentation.

### 3.3. Extraction and Purification

The marine-derived fungal strain F00785 was statically cultured on potato-dextrose-agar (PDA) medium at 28 °C for 18 days. The fermented material was diced and extracted with the mixed solvent of EtOAc/MeOH/AcOH (v/v/v, 80/15/5) (3 × 5 L). The organic solution was combined and concentrated in vacuum at 40 °C to yield crude syrup (49.2 g). The crude syrup was suspended in EtOAc and washed with H_2_O, then the EtOAc layer was concentrated and resuspended in MeOH and petroleum ether. The MeOH layer was concentrated to give the crude extract.

The crude extract (12.87 g) was fractionated by RP-18 column (160 g) using MeOH–H_2_O gradient (30%, 40%, 50%, and 100%) as the eluent to yield four fractions (Fraction I-IV). Fraction I (840.0 mg) was sequentially subjected to a Sephadex LH-20 (160 g) using MeOH as the mobile phase and a Sephadex LH-20 (160 g) using acetone as the eluent to yield two portions, part A (32.5 mg) and part B (78.9 mg). Part A was subjected to preparative TLC to yield **6** (4.0 mg). Part B was purified on silica column chromatography using PE (petroleum ether)/EtOAc (v/v, 3/1) as the eluent to obtain **8** (3.5 mg). Fraction II (339.0 mg) was subjected to Sephadex LH-20 (160 g) eluting with CH_2_Cl_2_/MeOH (v/v, 1/2) and then silica gel column eluting with PE/EtOAc (v/v, 1/2) to afford **2** (7.0 mg). Fraction IV (1.56 g) was sequentially subjected to a RP-18 column (80 g) eluting with a MeOH–H_2_O gradient (30%, 40%, 50%, and 100%), Sephadex LH-20 (160 g) eluting with CH_2_Cl_2_/MeOH (v/v, 1/2), Sephadex LH-20 (80 g) eluting with acetone, and preparative TLC to afford **1** (28.9 mg), **4** (10.0 mg), **5** (8.0 mg),**7** (38.0 mg), and **9** (4.5 mg). Compound **3** (2 mg) was obtained by further purification using semi-preparative HPLC (XDB C18 column, 10 × 250 mm, 5 μm, flow = 2 mL/min). The R_f_ values of compounds **4**–**9** on TLC over silica gel developed with CHCl_3_/MeOH (v/v, 10:1) are 0.85, 0.40, 0.40, 0.65, 0.35, and 0.35, respectively.

6-*O*-α-d-Ribosylasperentin (**1**). Pale yellow oil; ${\left[\text{α}\right]}_{\text{D}}^{20}$ = +122 (c = 0.7, MeOH), UV (MeOH) λ_max_ 265.9 and 302.0 nm; IR (KBr) ν_max_ 3364 and 1667 cm^−1^; ^1^H and ^13^C NMR, see Table 1; HR-ESI-MS *m/z* 447.1632 [M + Na]^+^ (calcd for C_21_H_28_O_9_Na, 447.1631).

6-*O*-α-d-Ribosyl-8-*O*-methylasperentin (**2**). Pale yellow oil; ${\left[\text{α}\right]}_{\text{D}}^{20}$ = +96 (*c* = 0.44, MeOH), UV (MeOH) λ_max_ 260.0 and 302.0 nm; IR (KBr) ν_max_ 3445 and 1700 cm^−1^; ^1^H and ^13^C NMR, see Table 1; HR-ESI-MS *m/z* 439.1975 [M + H]^+^ (calcd for C_22_H_31_O_9_, 439.1968).

5-Hydroxyl-6-*O*-methylasperentin (**3**). White needle; ${\left[\text{α}\right]}_{\text{D}}^{20}$ = −12 (*c* = 0.18, MeOH), m.p. 125.7 °C, UV (MeOH) λ_max_ 268.9 and 333.0 nm; IR (KBr) ν_max_ 3319 and 1657 cm^−1^; ^1^H and ^13^C NMR, see Table 1; HR-ESI-MS *m/z* 345.1308 [M + Na]^+^, (calcd for C_17_H_22_O_6_Na, 345.1314).

### 3.4. Acid Hydrolysis and Stereochemistry Determination of the Ribofuranose of 6-O-α-d-Ribosyl Asperentin (**1**)

A solution of **1** (90.0 mg) in 3 N HCl (60 mL) was stirred at 80 °C for 1 h. After the hydrolysis reaction was completed, the reaction mixture was concentrated under vacuum. The resulting residue was suspended in H_2_O (40 mL) and extracted with CH_2_Cl_2_ (3 × 40 mL). Then the aqueous layer was concentrated and dried under vacuum. The resulting residue was acetylated with Ac2O/Py at room temperature to yield an acetate product that was purified by Sephadex LH-20 (80 g) eluting with acetone/MeOH (v/v, 4/1) to afford a tetra-acetate of ribofuranose (4.1 mg). The specific rotation of this tetra-acetate product, ${\left[\text{α}\right]}_{\text{D}}^{20}$ = −17 (*c* = 0.68, MeOH), is consistent with the reported value of d-ribose [20]. ^1^H-NMR (600 MHz, CDCl_3_) δ 6.06 (1H, d, *J* = 4.9 Hz, H-1), 5.06 (1H, d, *J* = 3.6 Hz, H-2), 5.51 (1H, brs, H-3), 5.18 (1H, d, *J* = 3.2 Hz, H-4), 3.94 (1H, dd, *J* = 12.5, 5.9 Hz, H_a_-5), 4.05 (1H, dd, *J* = 12.5, 3.0 Hz, H_b_-5), 2.16 (3H, s, Ac), 2.15 (3H, s, Ac), 2.13 (3H, s, Ac), 2.12 (3H, s, Ac). ^13^C-NMR (150 MHz, CDCl_3_) δ 90.9 (C-1), 67.3 (C-2), 66.2 (C-3), 66.3 (C-4), 62.7 (C-5), 169.9 (CH_3_CO), 169.8 (CH_3_CO), 169.5 (CH_3_CO), 168.8 (CH_3_CO), 20.9 (CH_3_CO), 20.8 (CH_3_CO), 20.7 (CH_3_CO), 20.7 (CH_3_CO).

### 3.5. Anti-Crop Pathogens Test

Anti-crop pathogen activity against *C. gleosporioides* Penz, *C. gleosporioides* (Penz) Sacc., and *B. cinerea* Pers was evaluated using a filter-paper disk method. The tested strains were cultivated on PDA plates at 28 °C. Compounds **1**–**9** were tested at a concentration of 5 mg/mL in MeOH using amphotericin B (5 mg/mL in DMSO) as the positive control. The tested compound solutions (5 μL) were transferred onto filtering paper disks (Φ = 5 mm) placed in the center of assay plates. After 48 h incubation at 28 °C, the diameters of inhibition zones were measured to evaluate the activity, a bigger size of the diameter indicated a stronger inhibition. All experiments were repeated in triplicate.

## 4. Conclusion

Three new compounds, 6-*O*-α-d-ribosylasperentin (**1**), 6-*O*-α-d-ribosyl-8-*O*-methylasperentin (**2**) and 5-hydroxyl-6-*O*-methylasperentin (**3**), along with six known asperentin derivatives (**4**–**9**), isolated and identified from the fermentation products of a halotolerant fungus *Aspergillums* sp. strain F00785 from the inner tissue of the marine alga. **1** and **2**, were found to be the first members with d-ribofuranose via α-glycosidic linkage. To the best of our knowledge, the α-d-ribofuranosyl moiety is unusual in metabolites from fungi, with only four natural products containing α-d-ribofuranosyl moiety reported up to now [19,21,23,24]; their functions in microorganisms need to be further investigated. Among the nine compounds, compound **4** exhibited strong inhibitory activity against three crop pathogens. Preliminary study of the structure activity relationships (SARs) of these nine compounds indicated that free hydroxyls at C-6 and C-8 are critical to the antifungal activity, while the hydroxyl substitution at the 4′ or 5′ position of the pyranoid ring is not favorable. In summary, discovery of these new secondary metabolites suggest that chemical investigations of marine microorganisms in relatively uninvestigated environments may provide significant natural chemical diversity for drug discovery.

## Supplementary Files

Supplementary File 1
