# Supplementary material for: Three New Asperentin Derivatives from the Algicolous Fungus Aspergillus sp. F00785
Source: Mar Drugs. 2014 Dec 15;12(12):5993–6002. doi: 10.3390/md12125993 (PMC4278214; doi:10.3390/md12125993)
Supplement: Supplementary File 1 [file marinedrugs-12-05993-s001.pdf]

Supplementary Information

Figure S1. The H spectrum of compound **1** (Tested in the first time, and the weigh may be too heavy resulting in the weak split peaks).

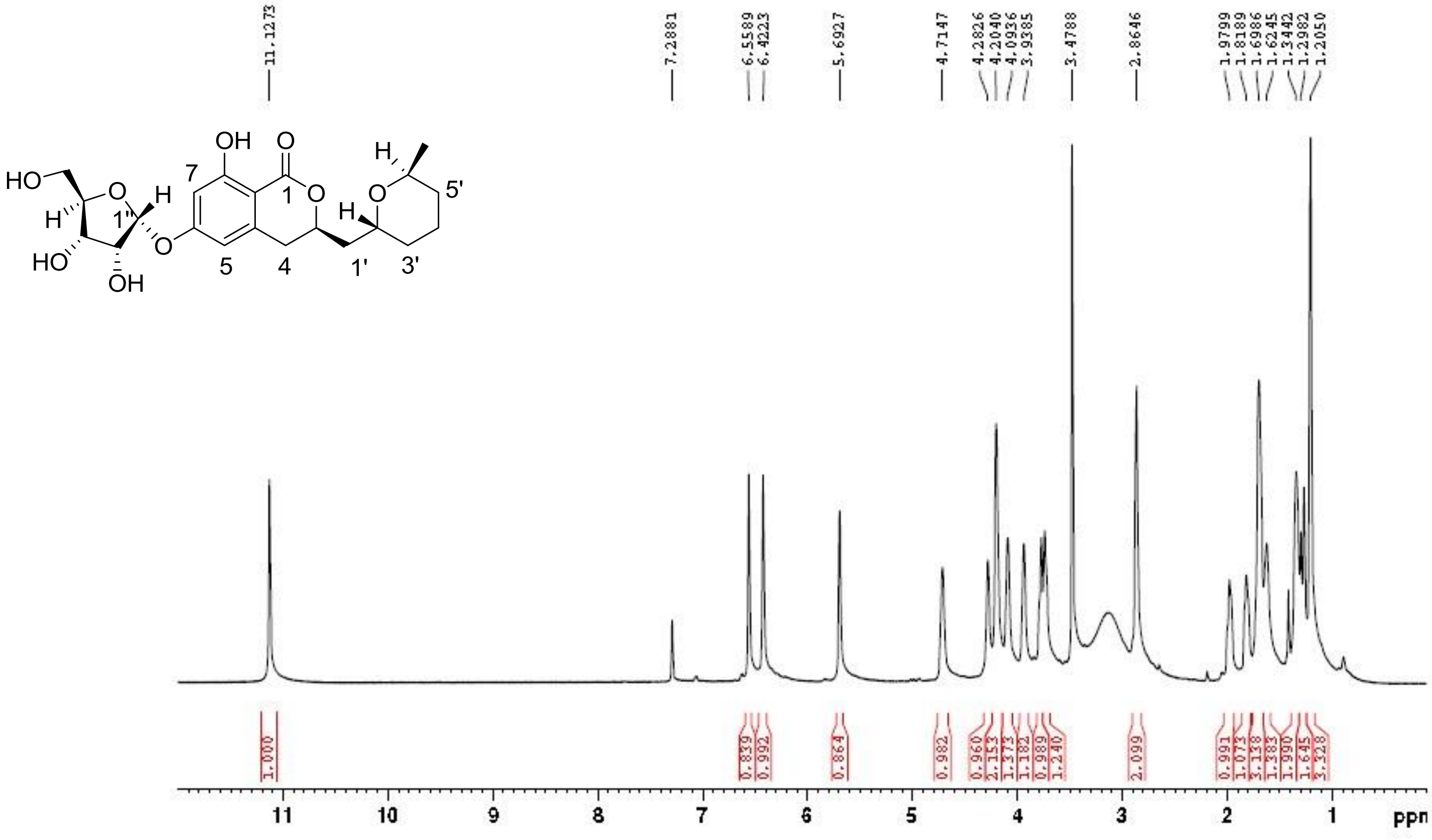

Figure S2. The H spectrum of compound **1**. (tested in the second time).

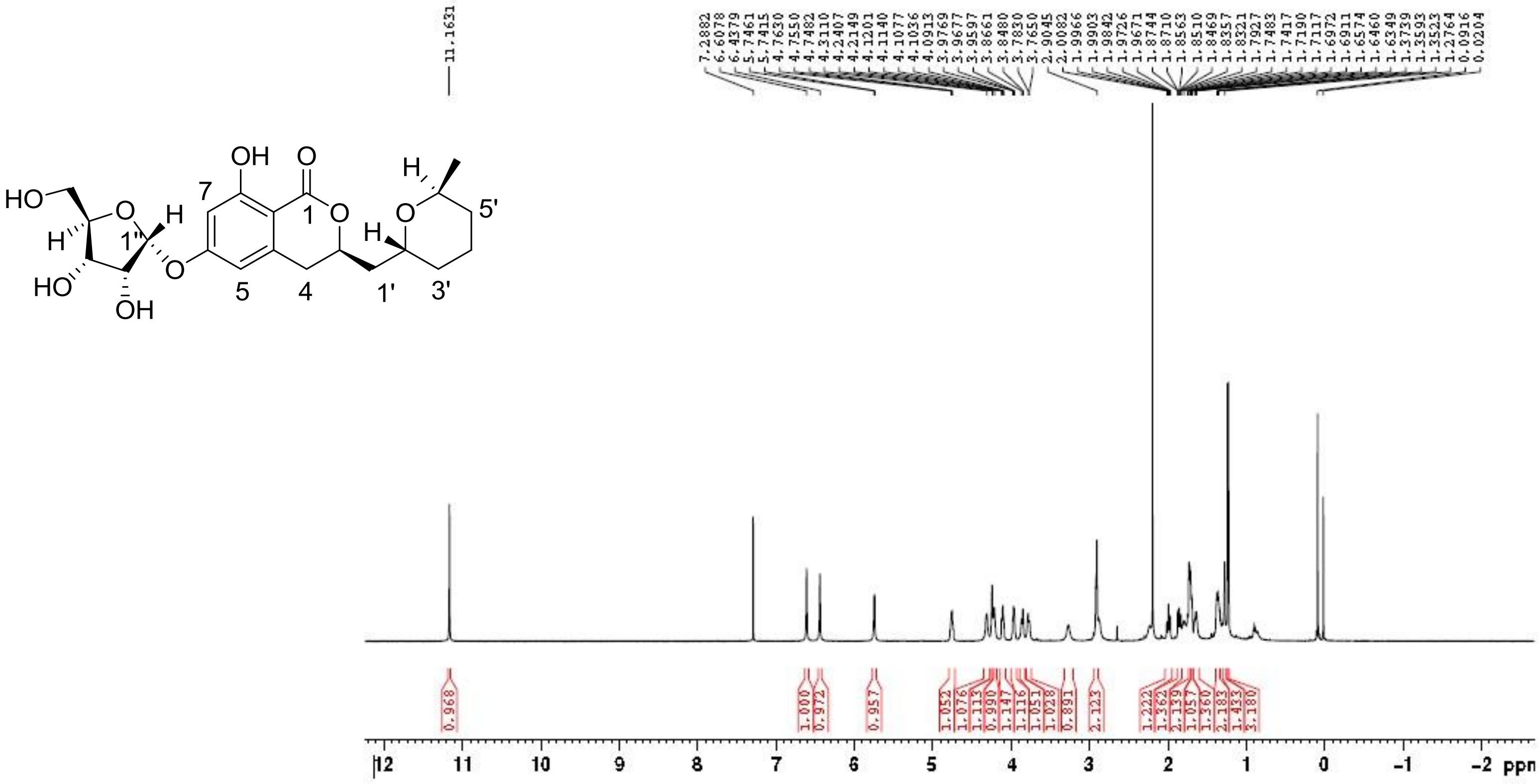

Figure S3. The C spectrum of compound **1**.

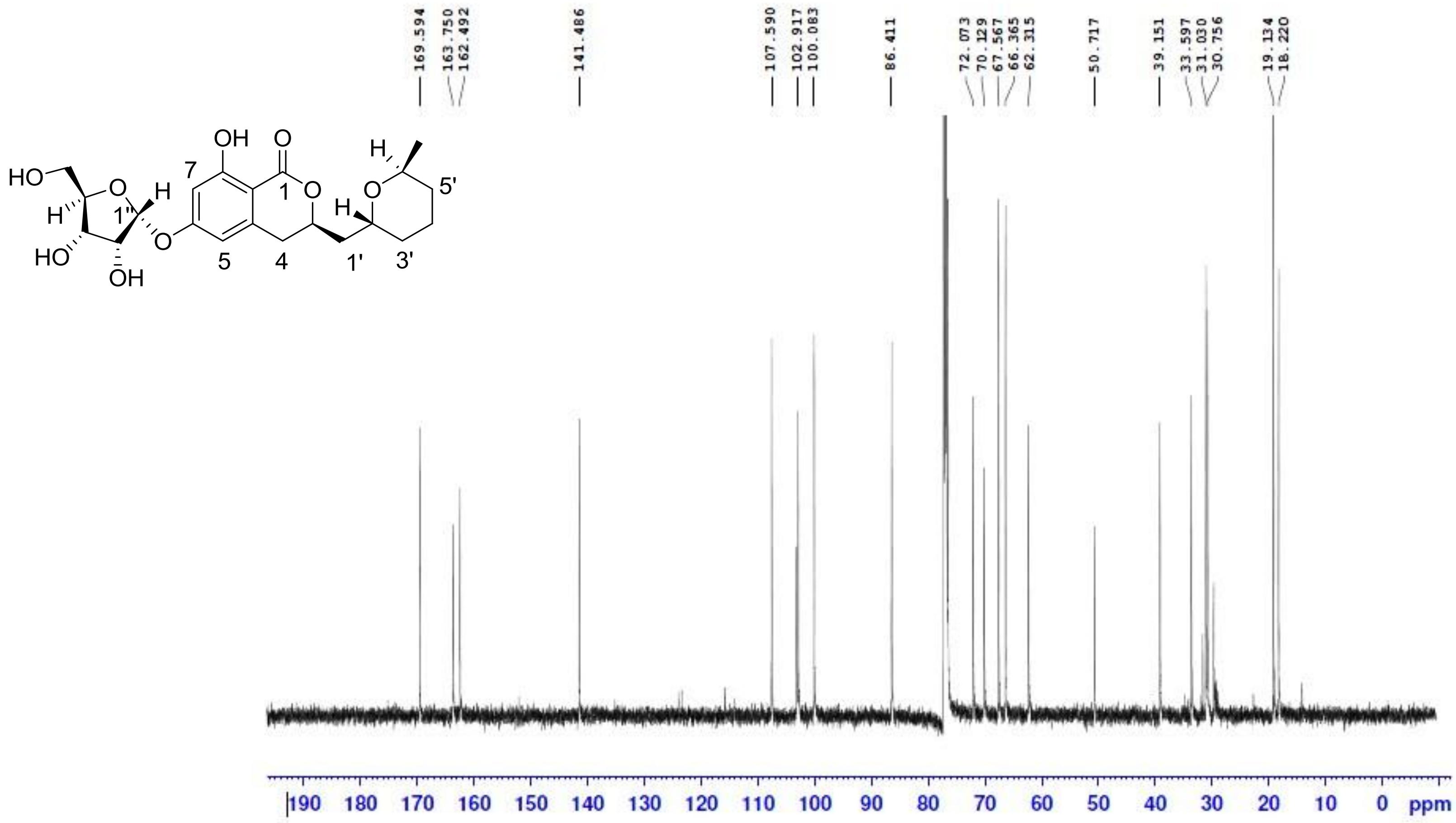

Figure S4. The QC spectrum of compound **1**.

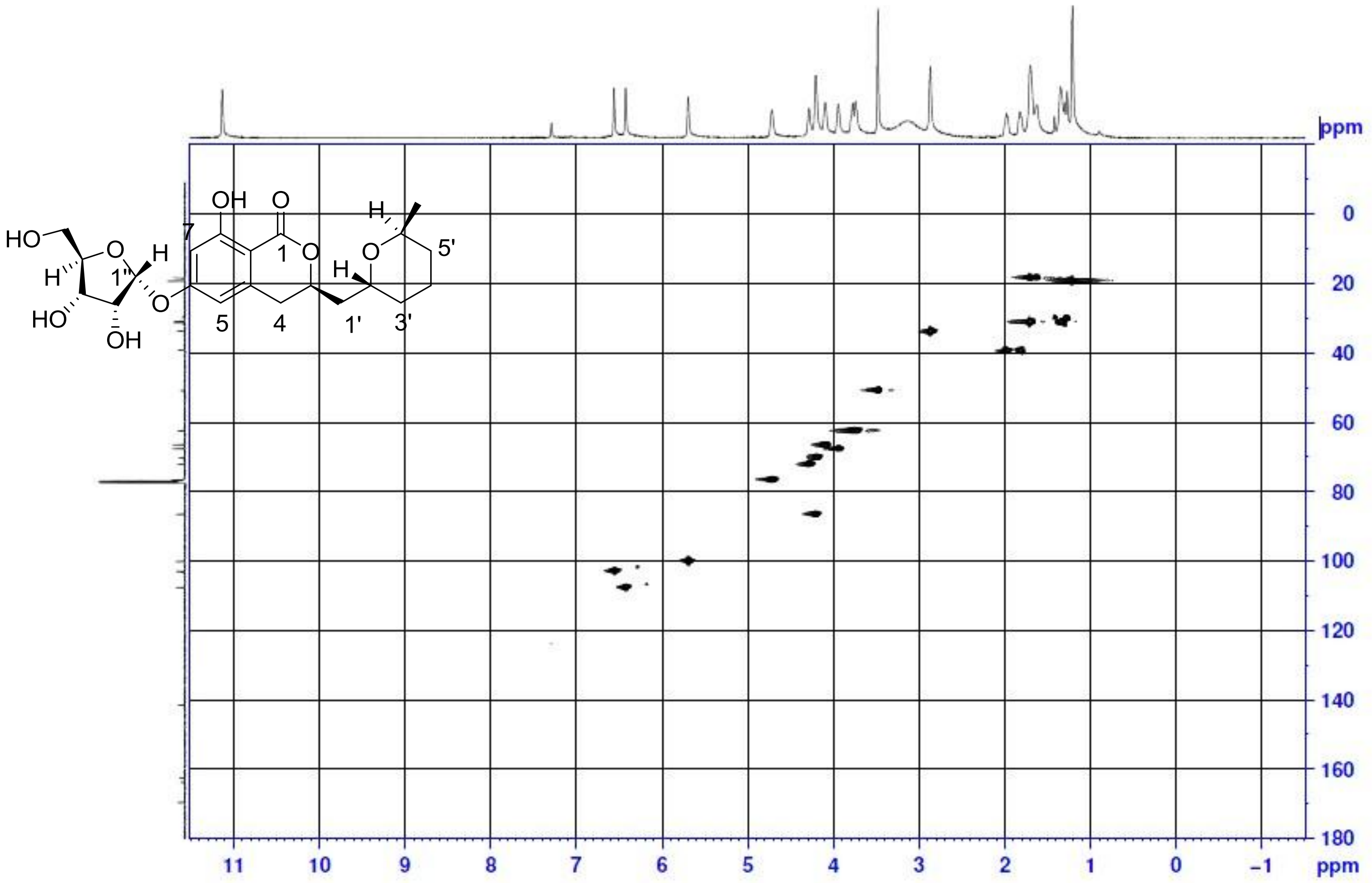

Figure S5. The HMBC spectrum of compound **1**.

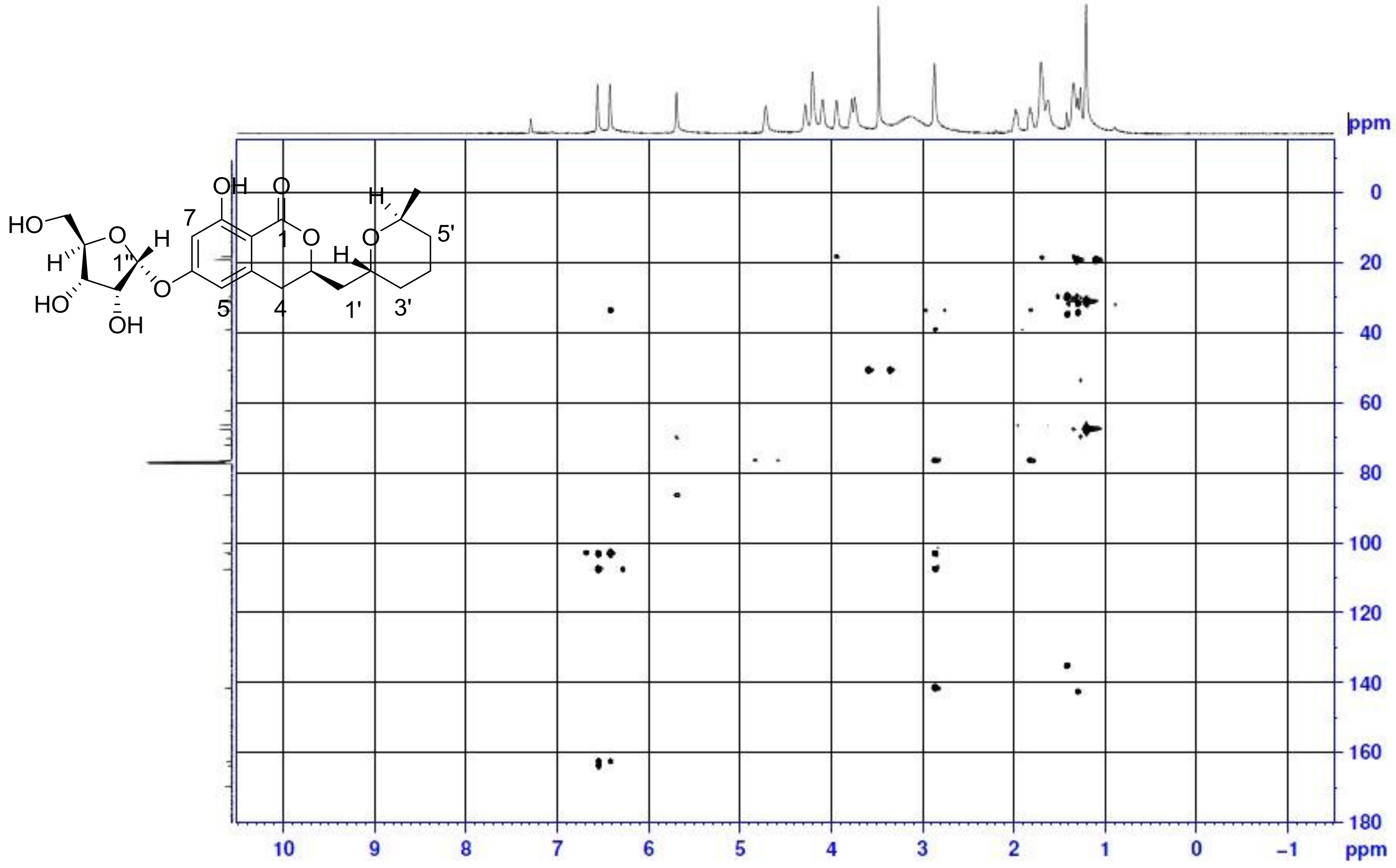

Figure S6. The COSY spectrum of compound **1**.

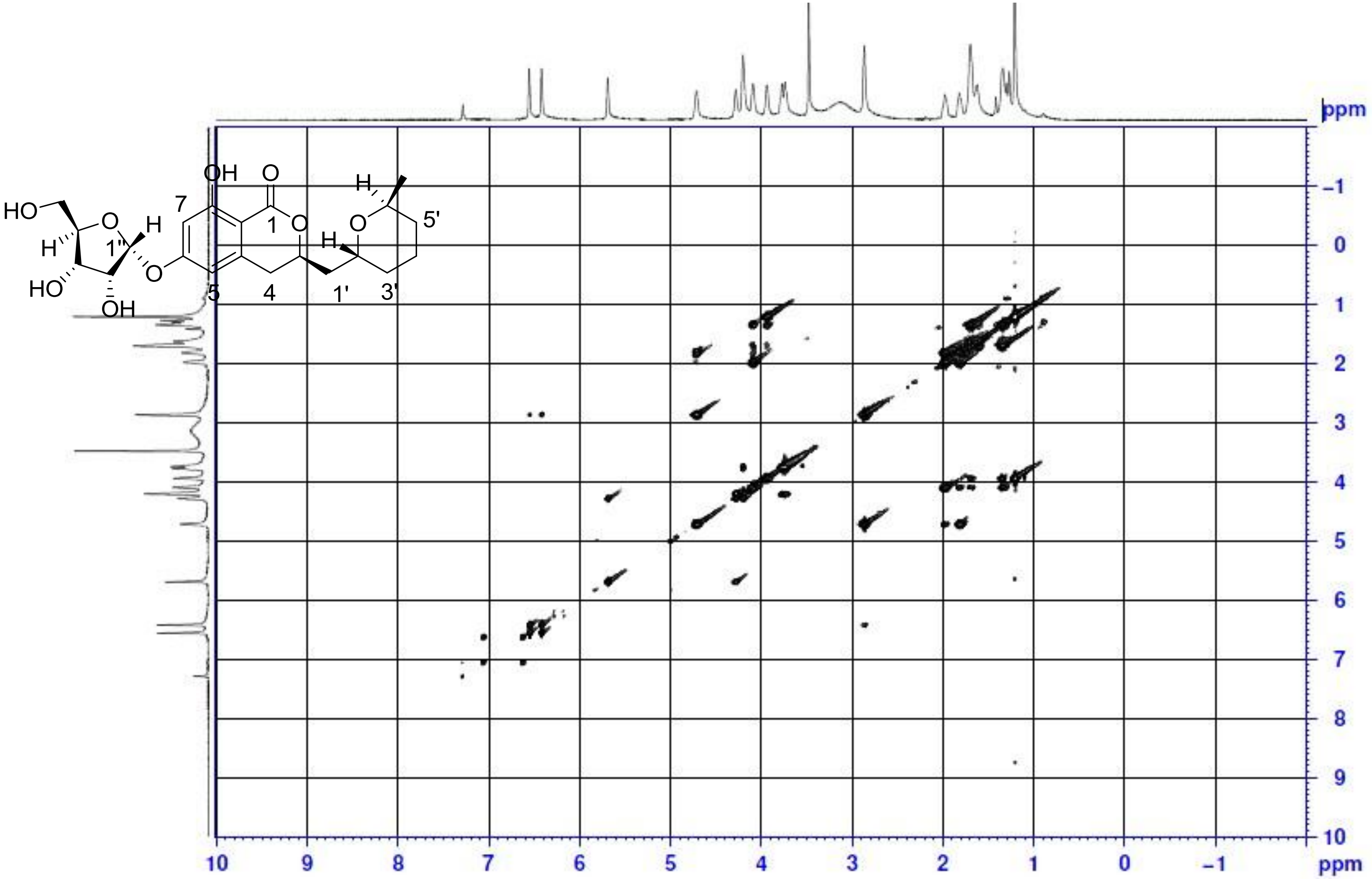

Figure S7. The NOE spectrum of compound **1**.

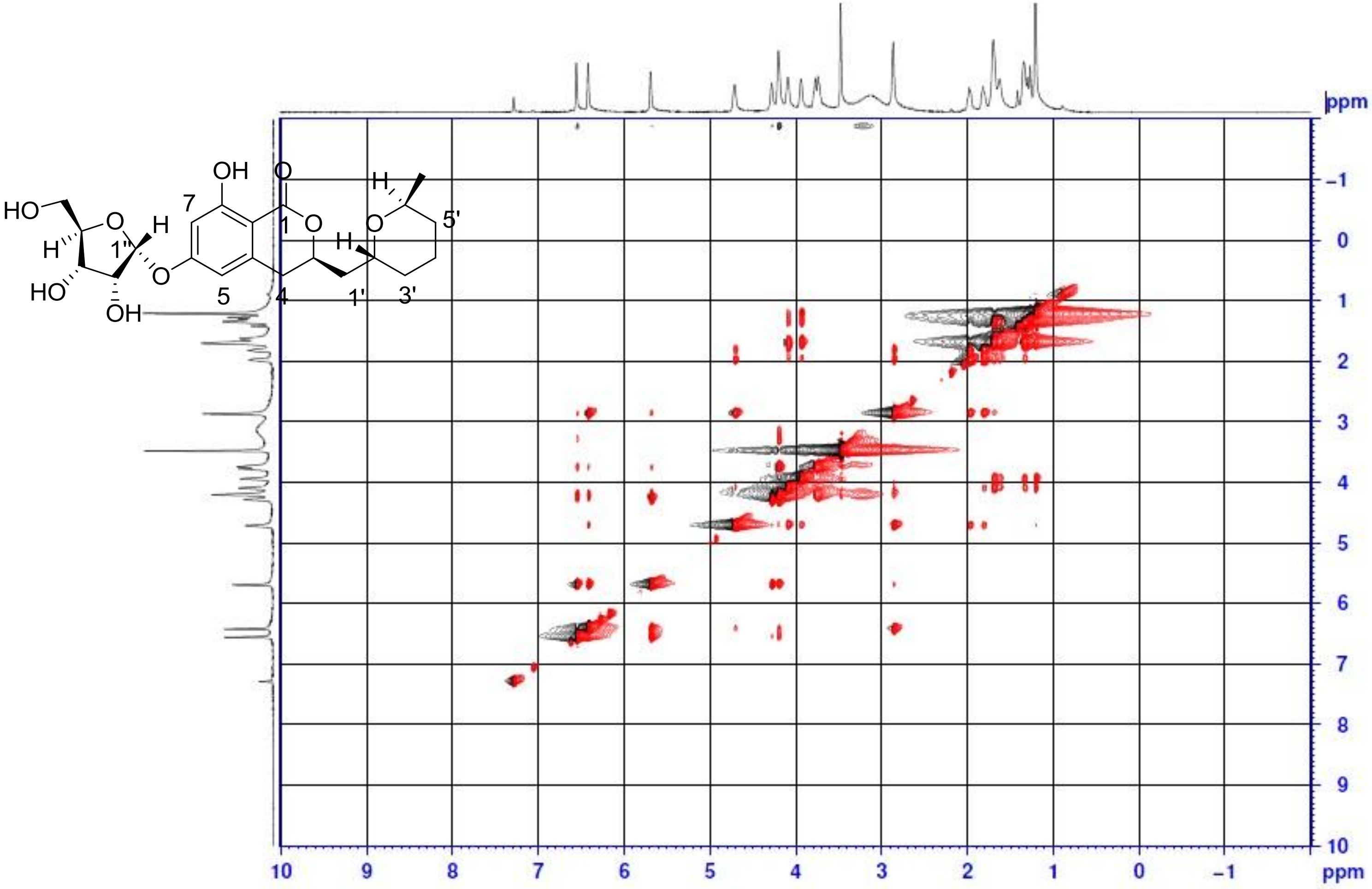

Figure S8. The H spectrum of compound **2**.

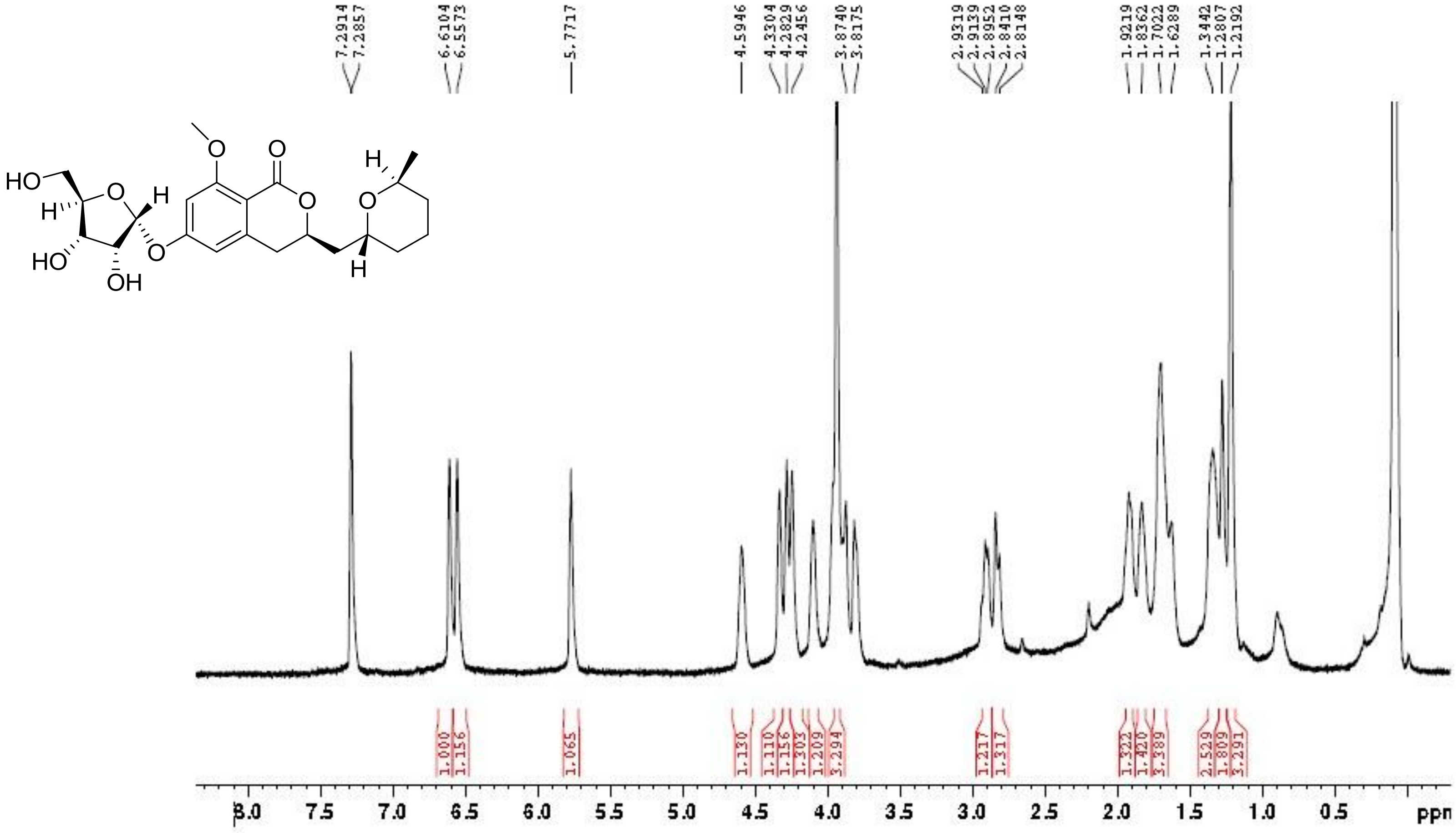

Figure S9. The C spectrum of compound **2**.

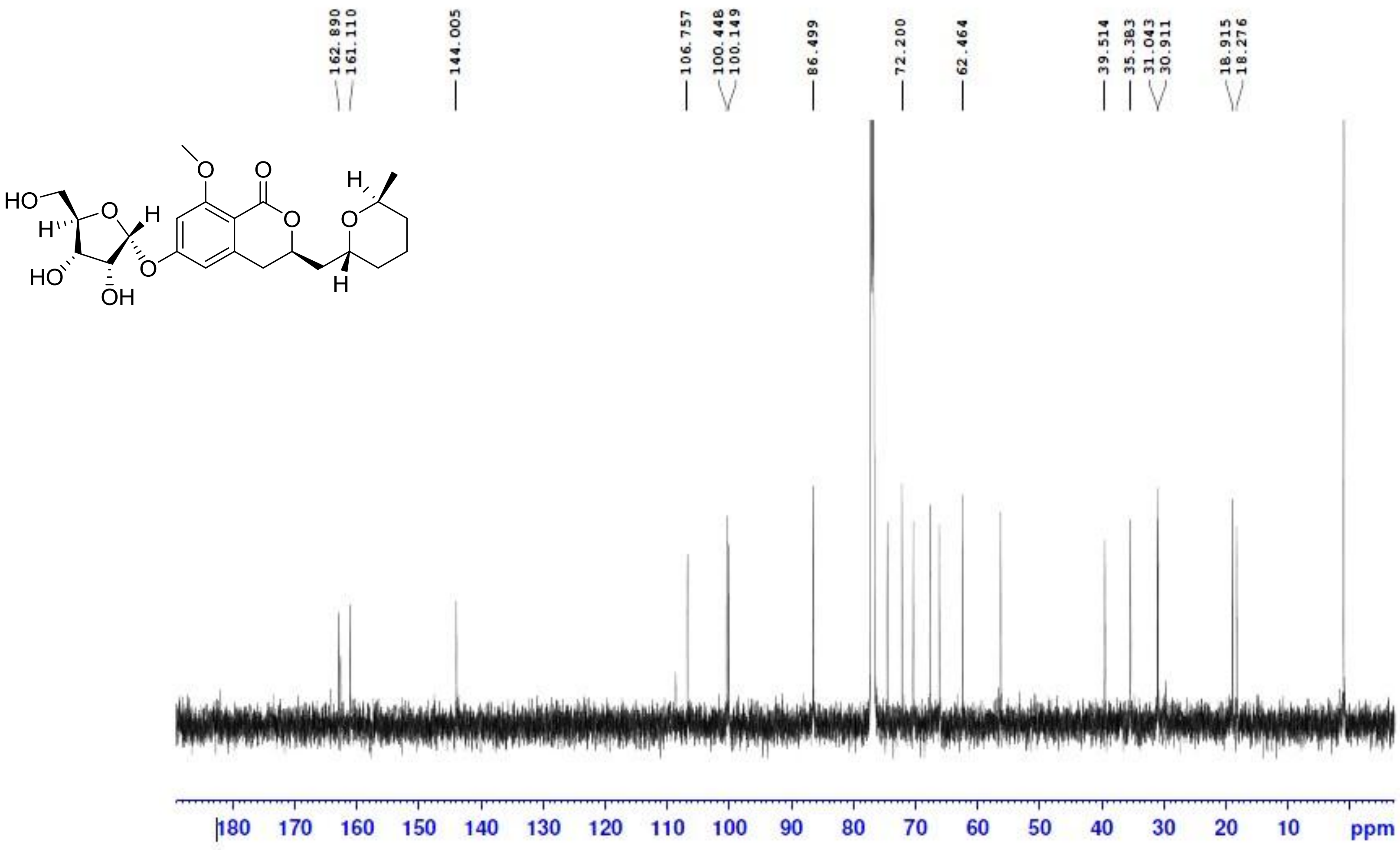

Figure S10. The H spectrum of compound **3**.

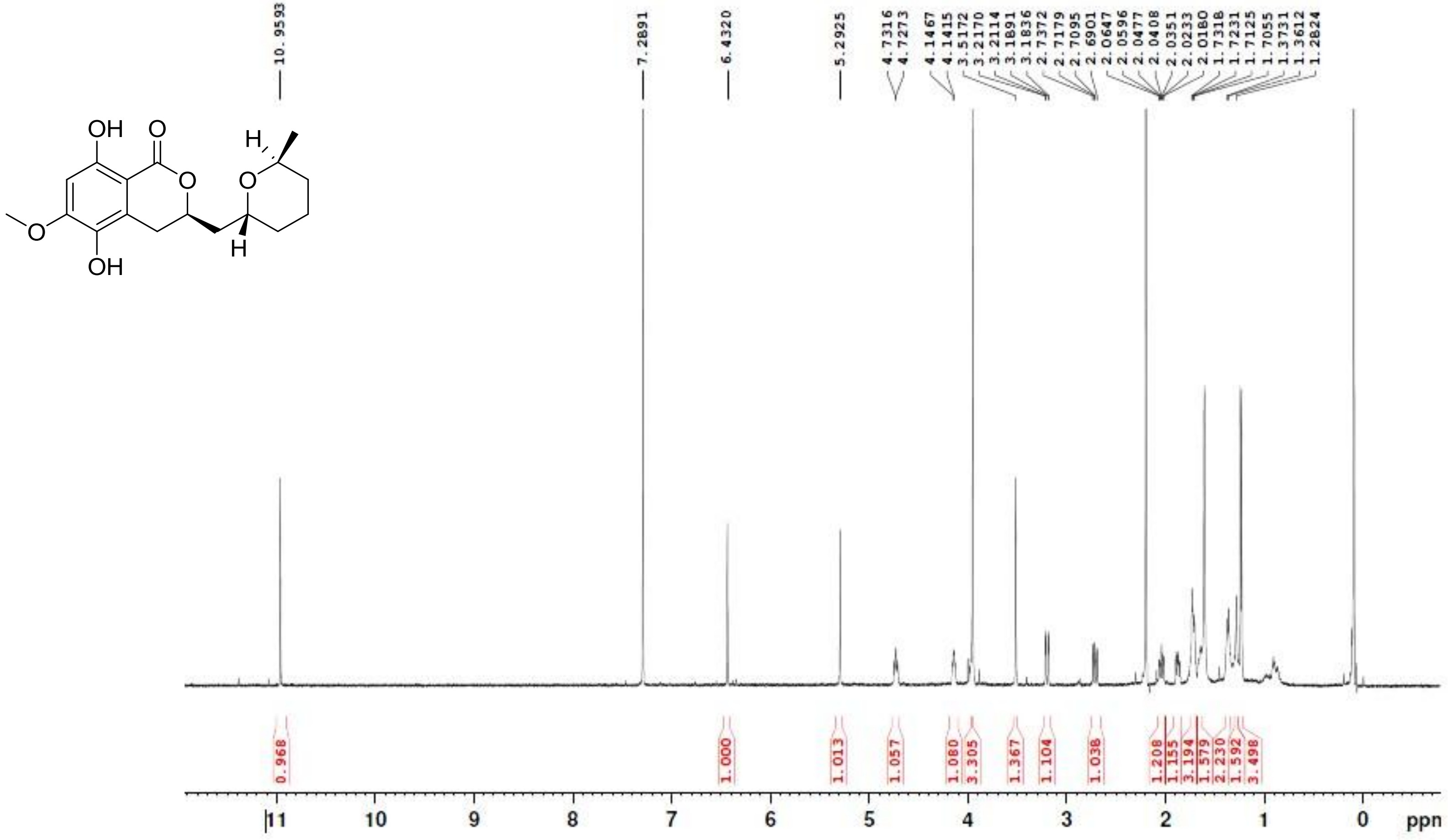

Figure S11. The C spectrum of compound **3**.

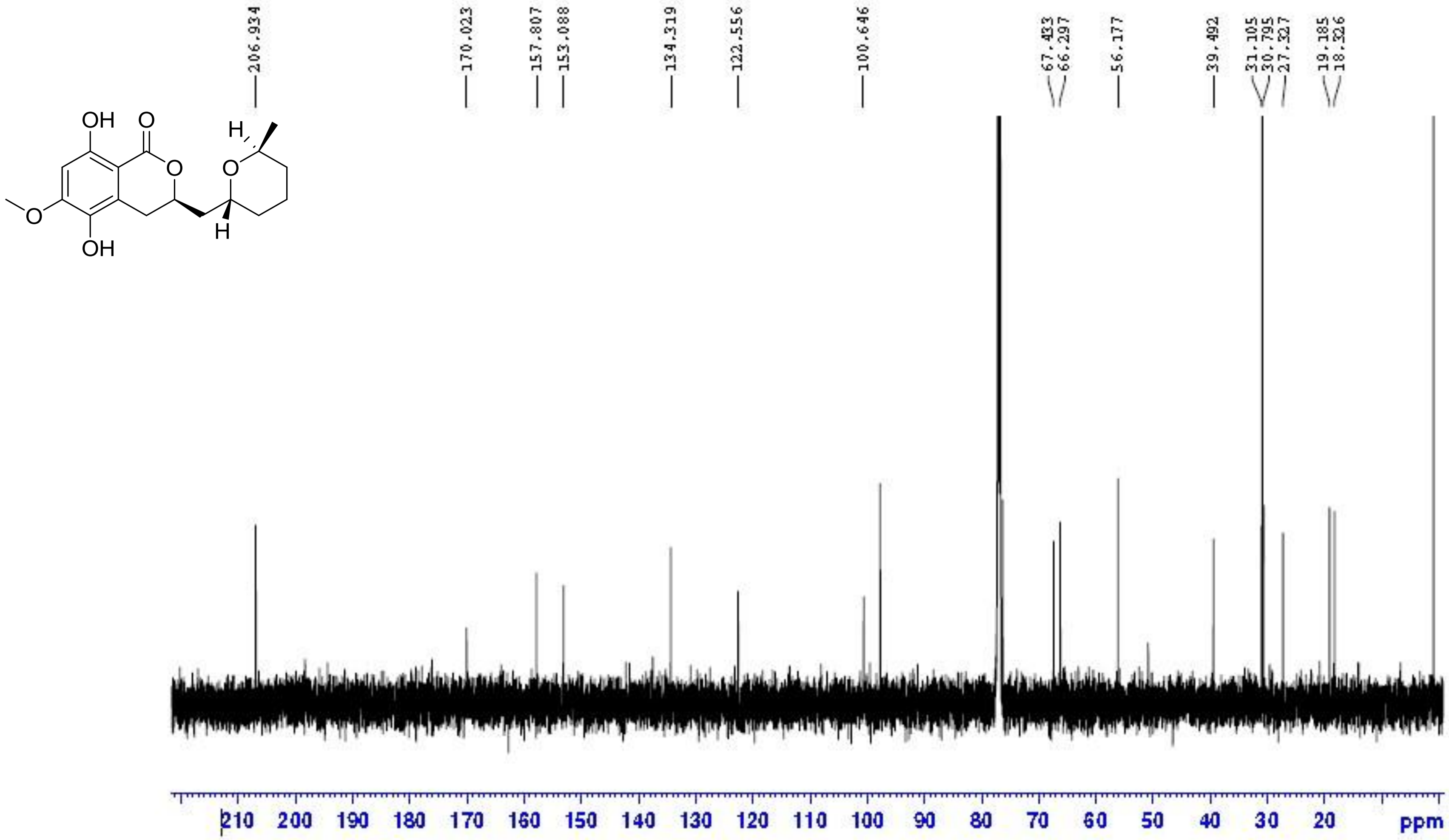

Figure S12. The H spectrum of compound **4**.

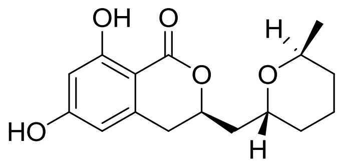

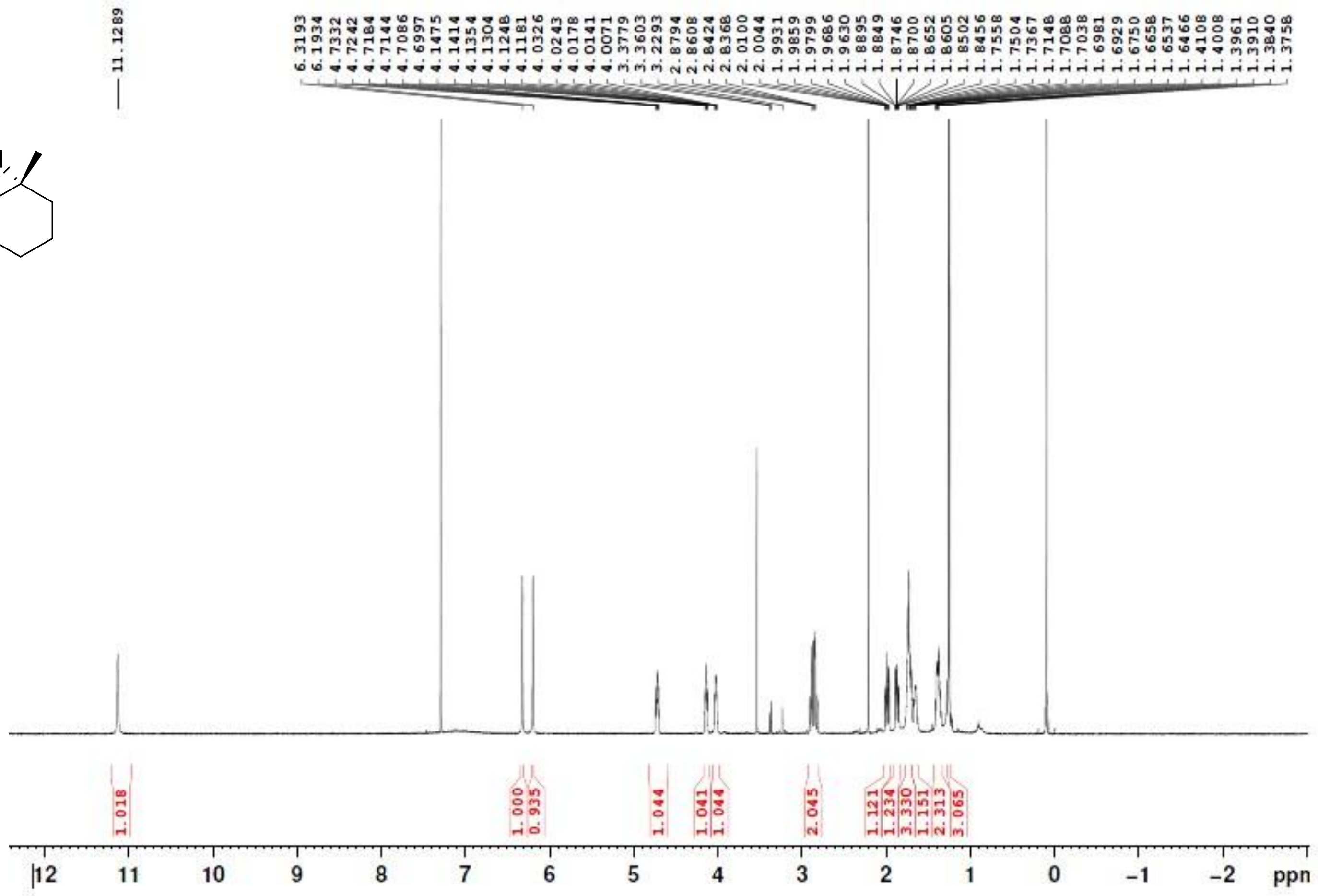

Figure S13. The C spectrum of compound **4**.

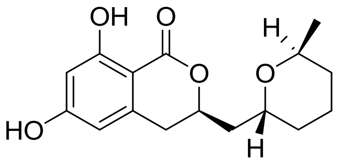

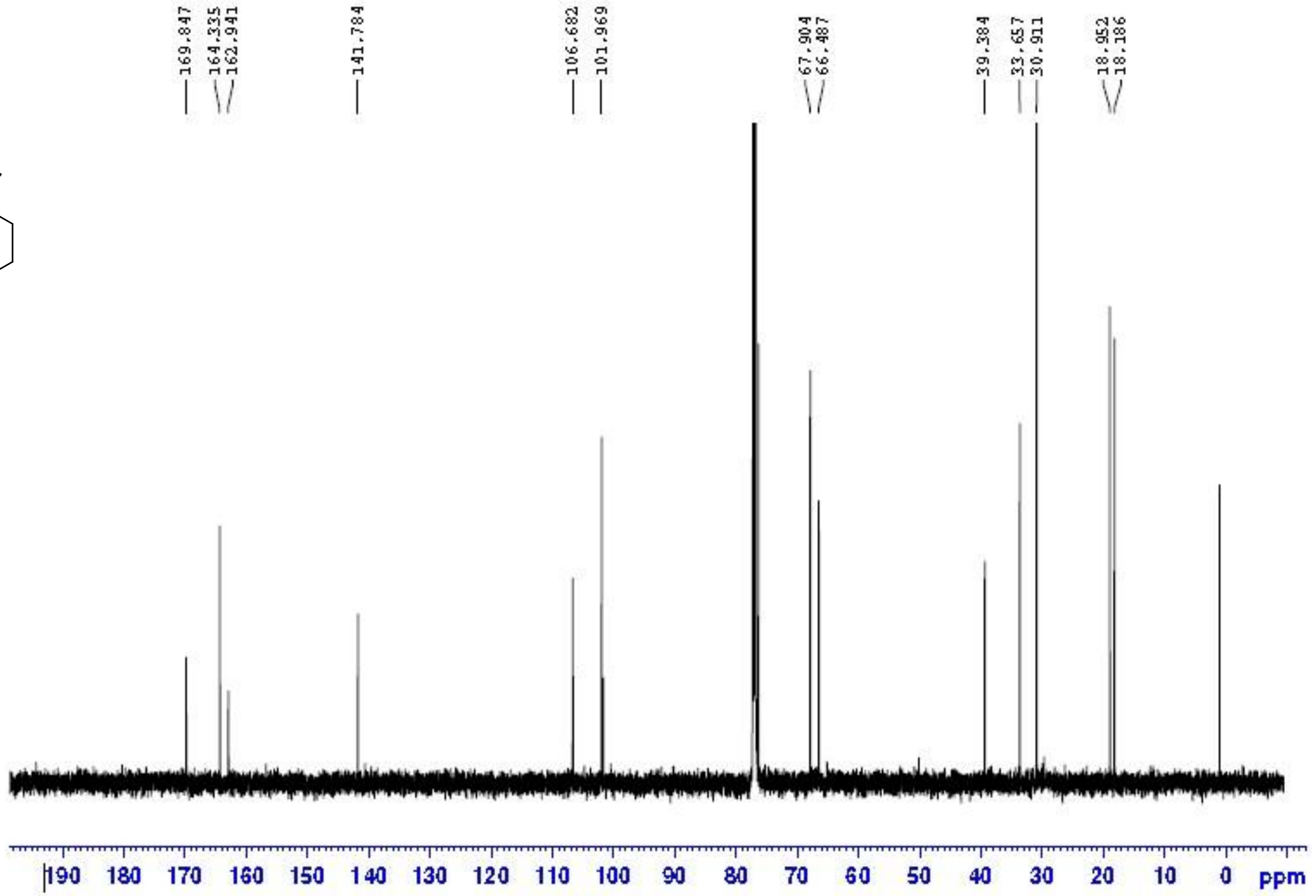

Figure S14. The H spectrum of compound **5**.

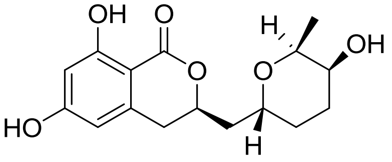

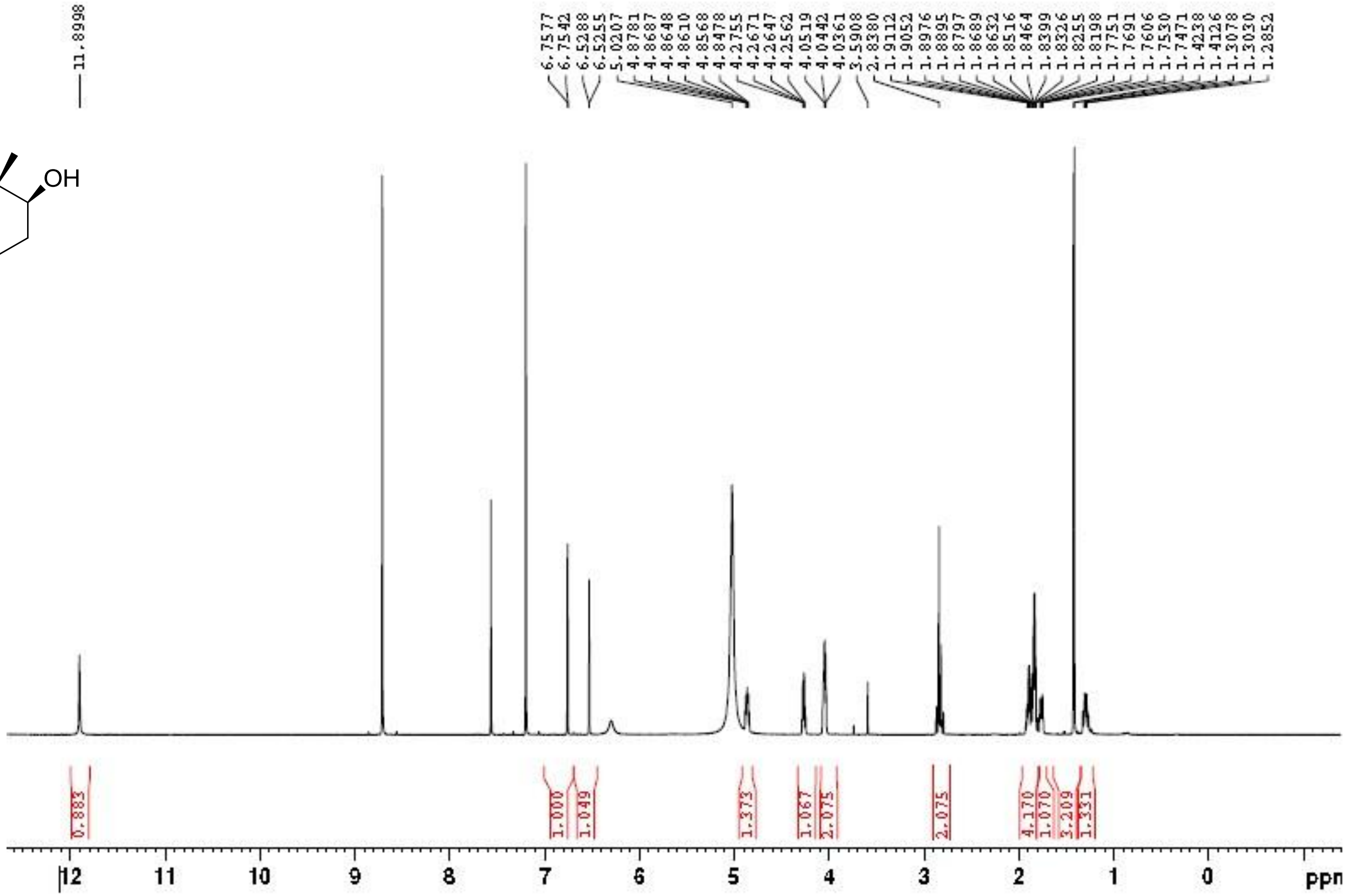

Figure S15. The C spectrum of compound **5**.

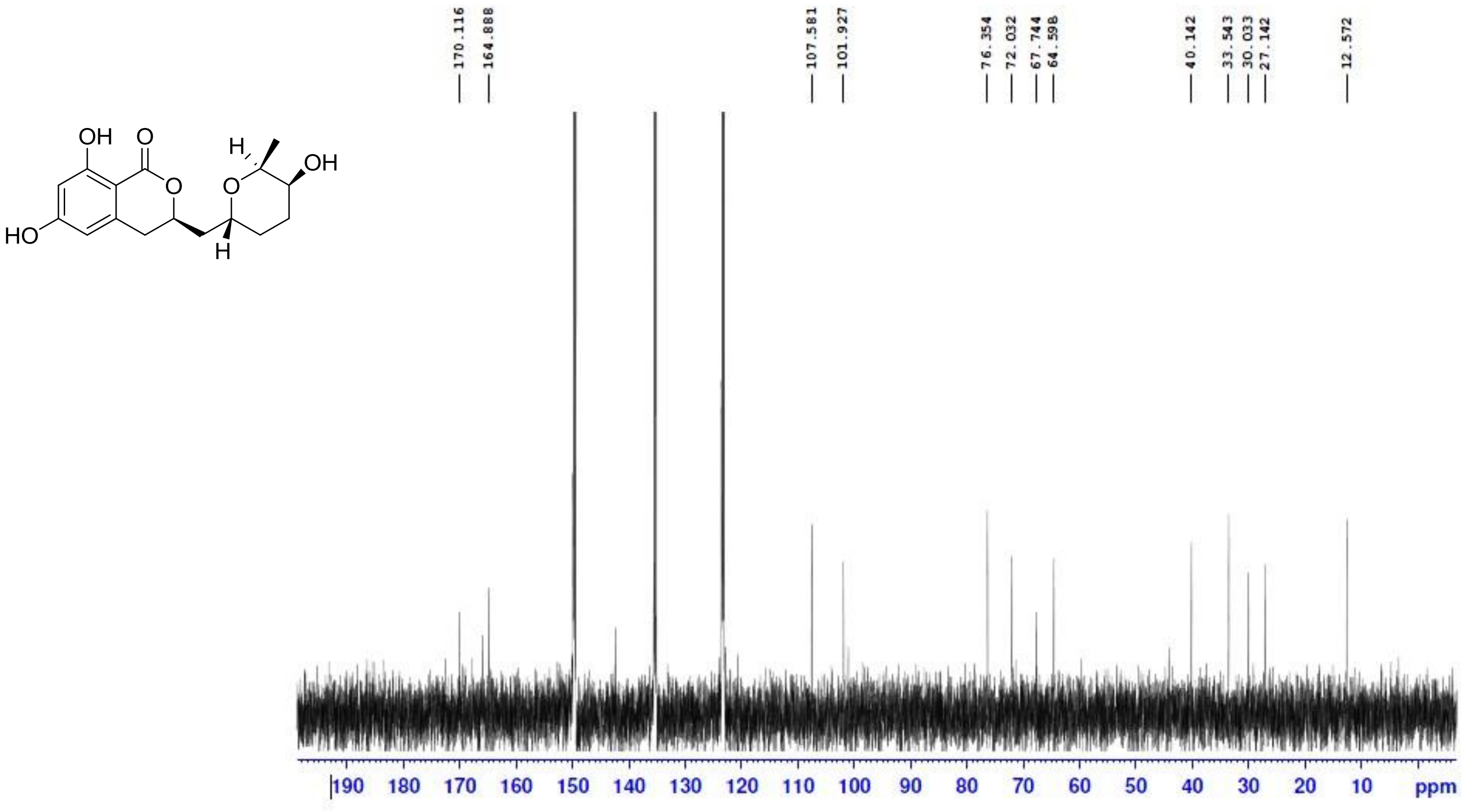

Figure S15. The H spectrum of compound **6**.

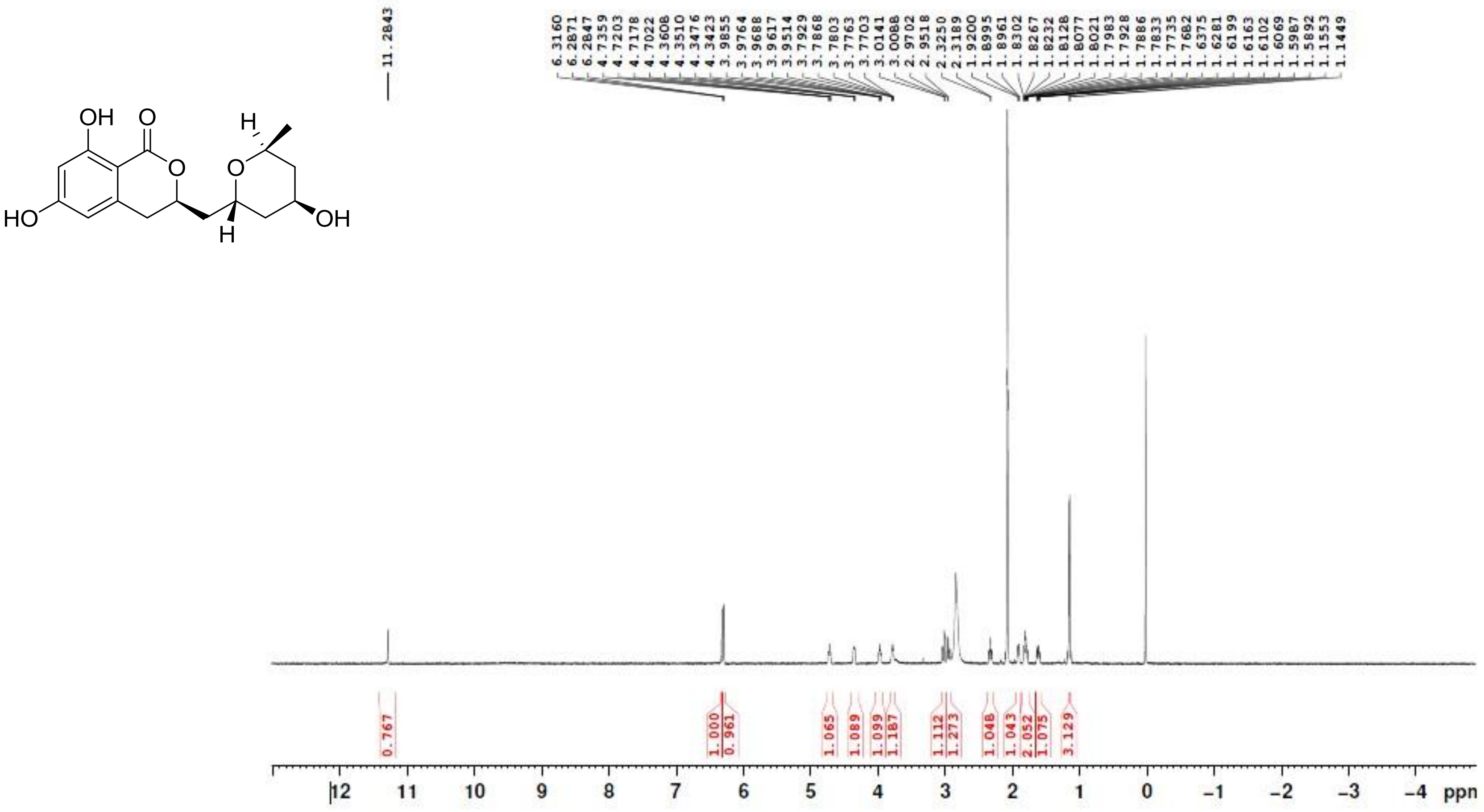

Figure S17. The C spectrum of compound **6**.

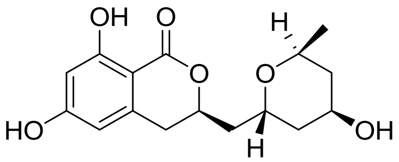

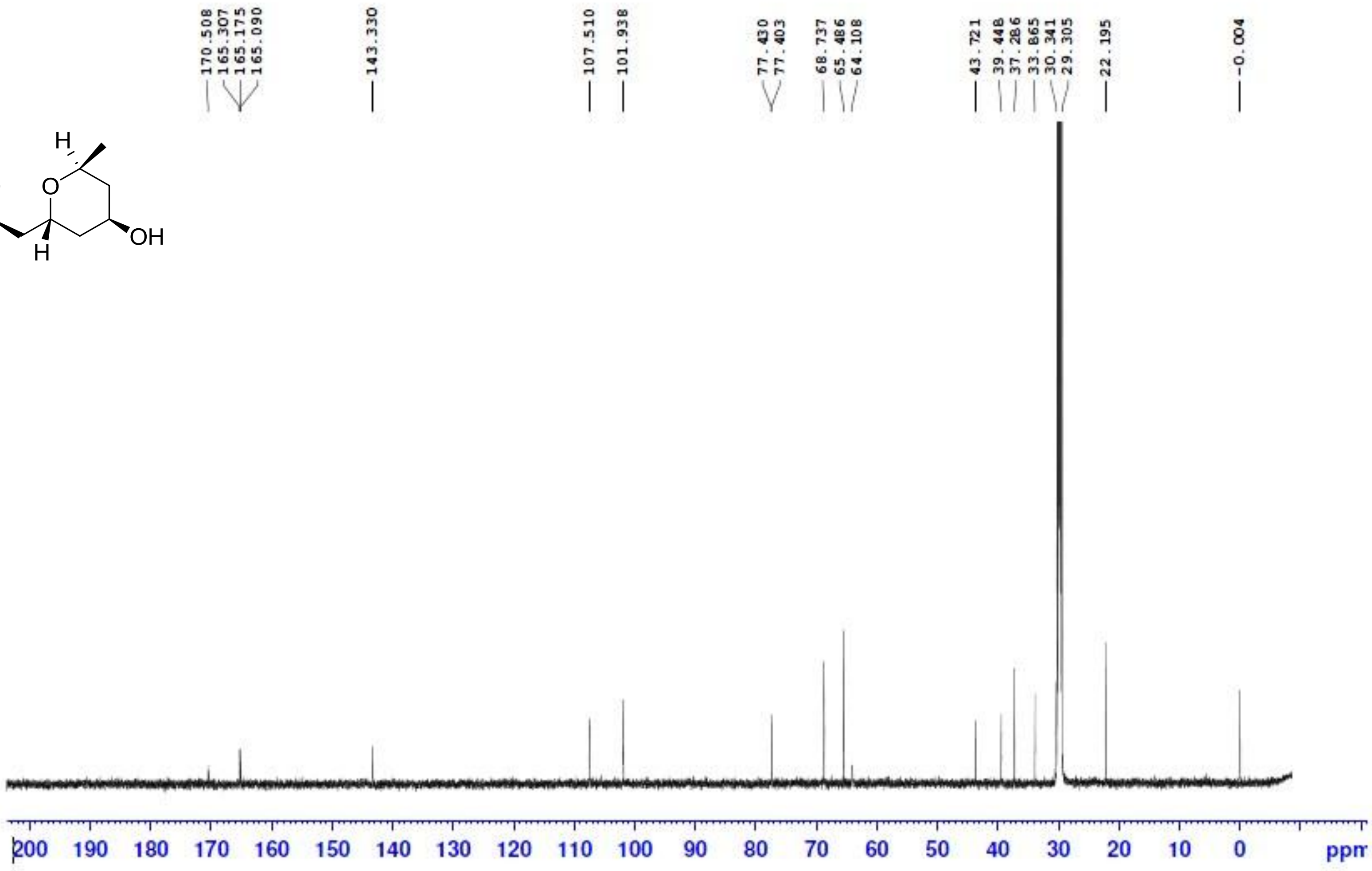

Figure S18. The H spectrum of compound **7**.

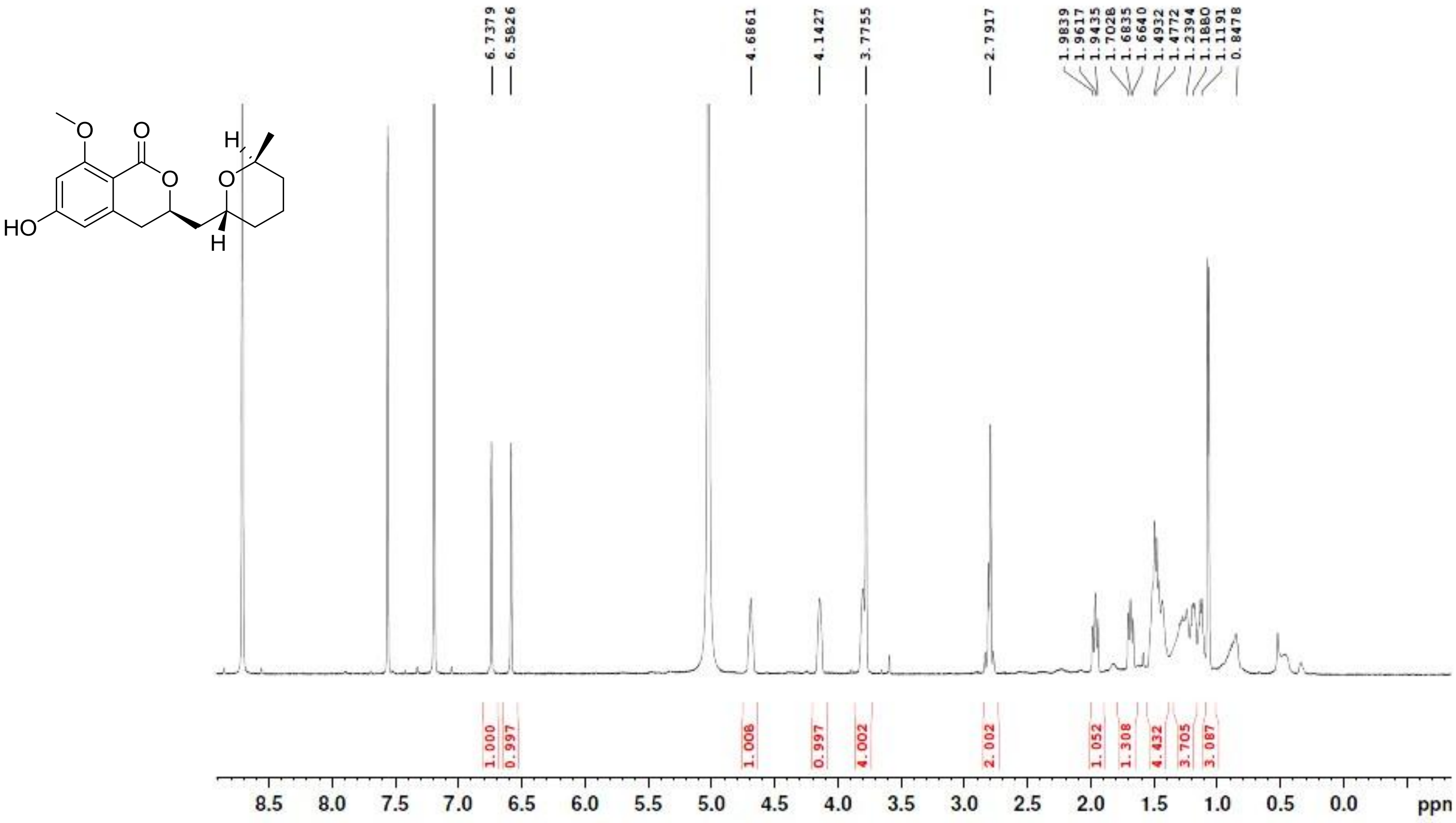

Figure S19. The C spectrum of compound **7**.

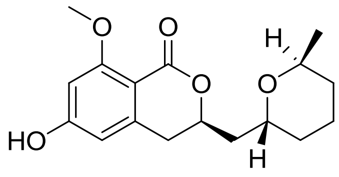

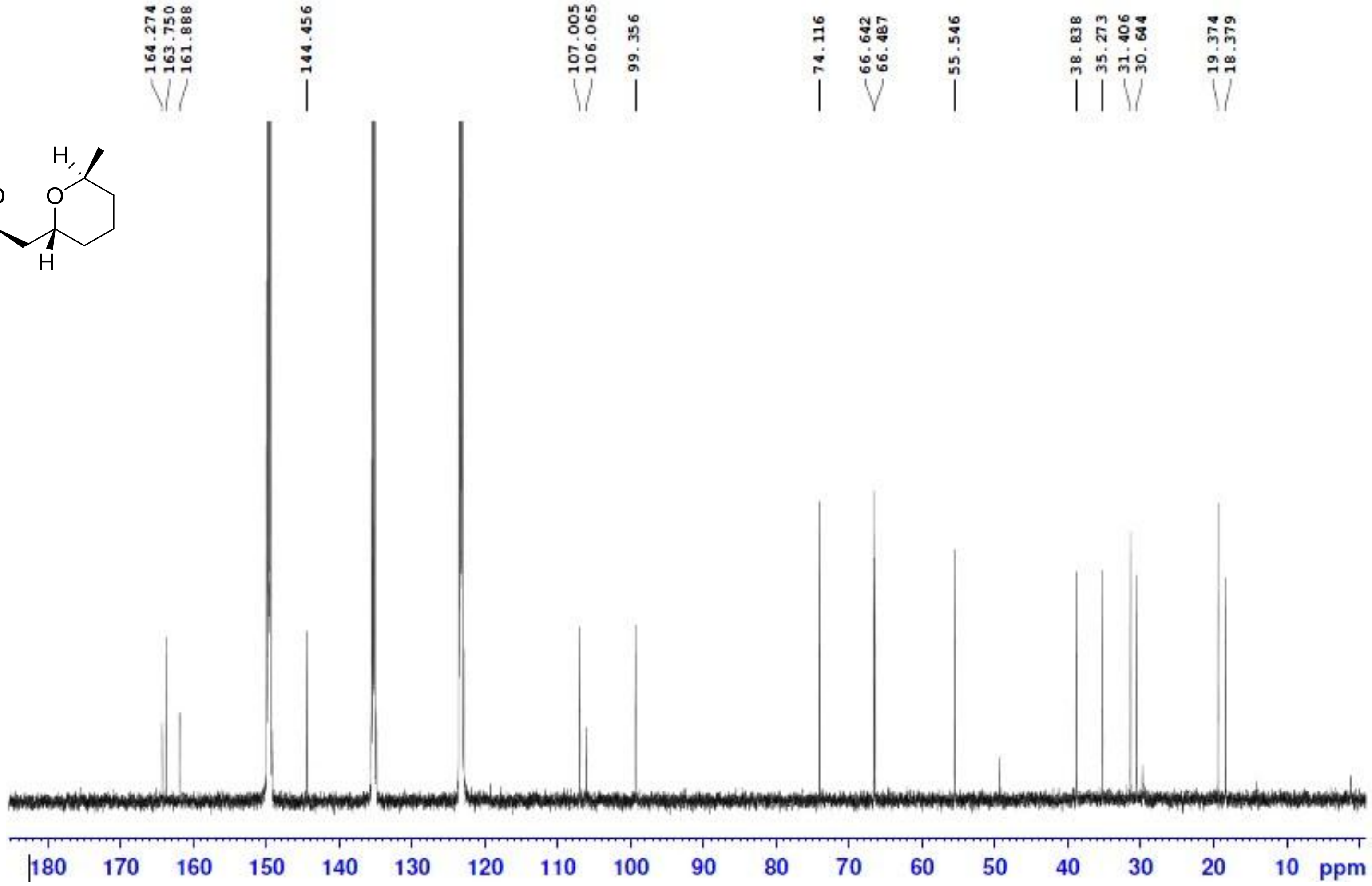

Figure S20. The H spectrum of compound **8**.

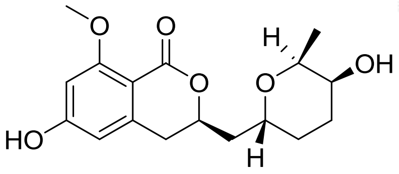

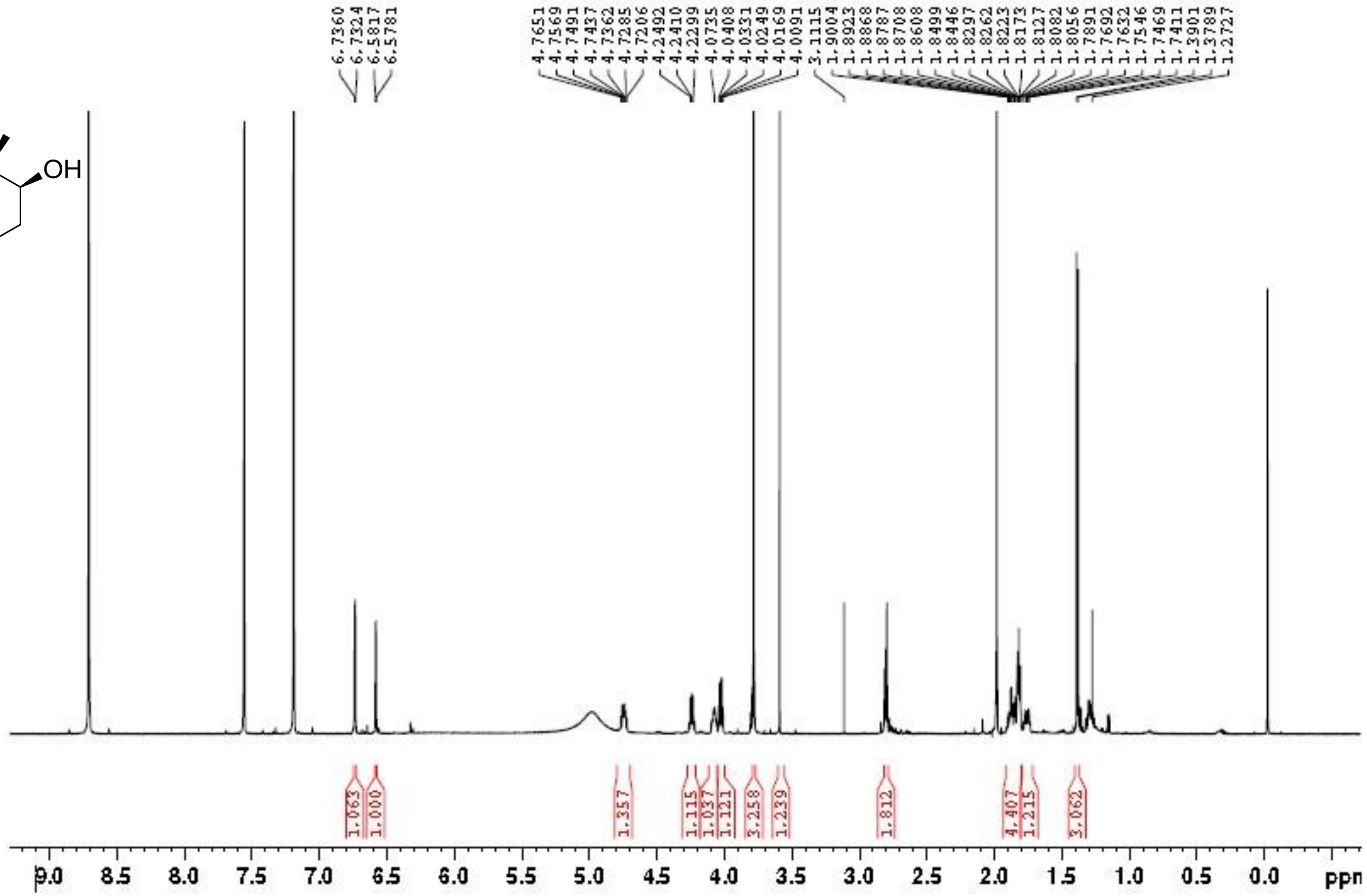

Figure S21. The C spectrum of compound **8**.

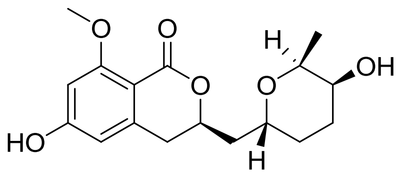

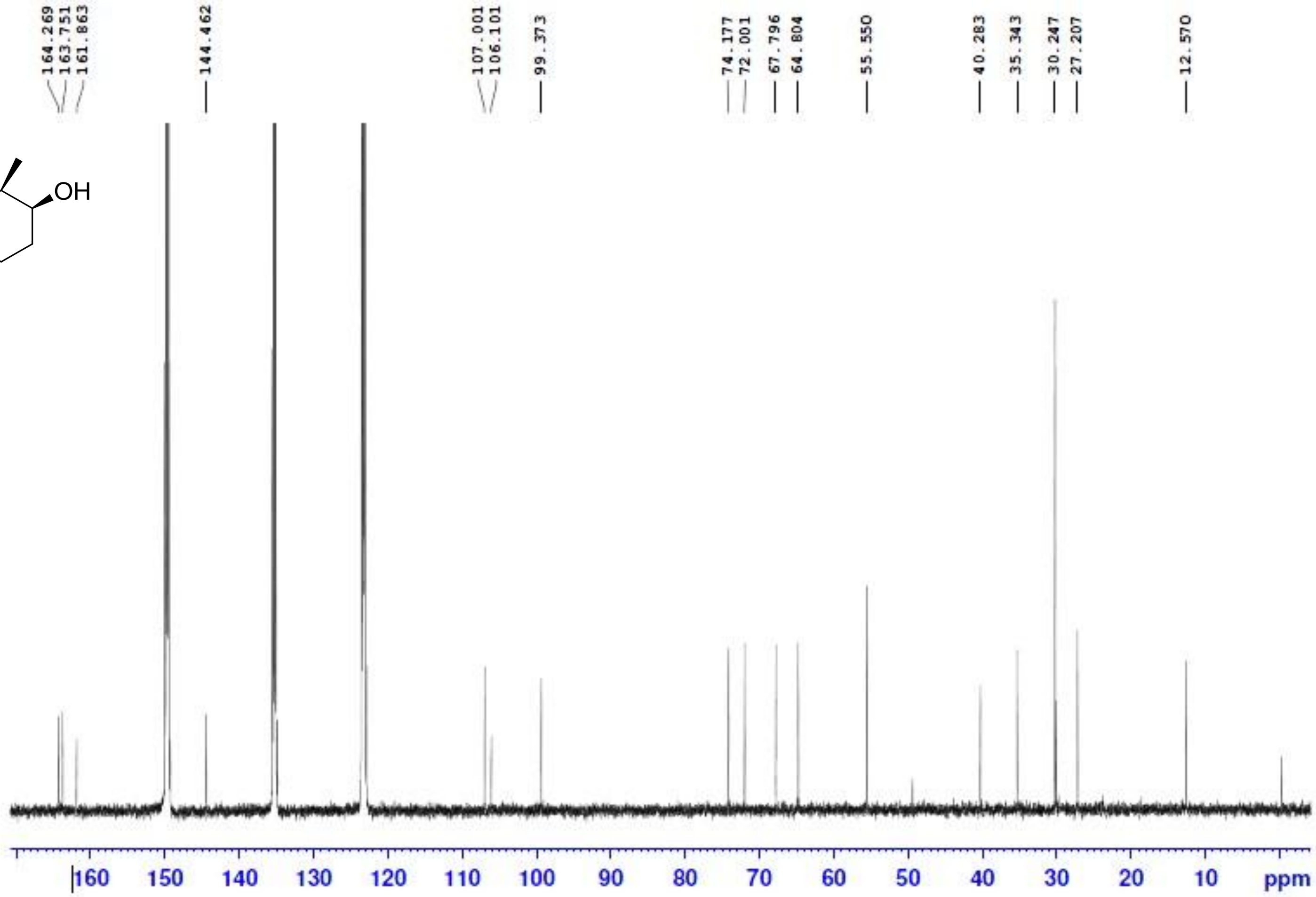

Figure S22. The H spectrum of compound **9**.

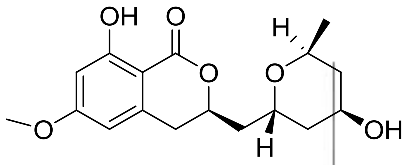

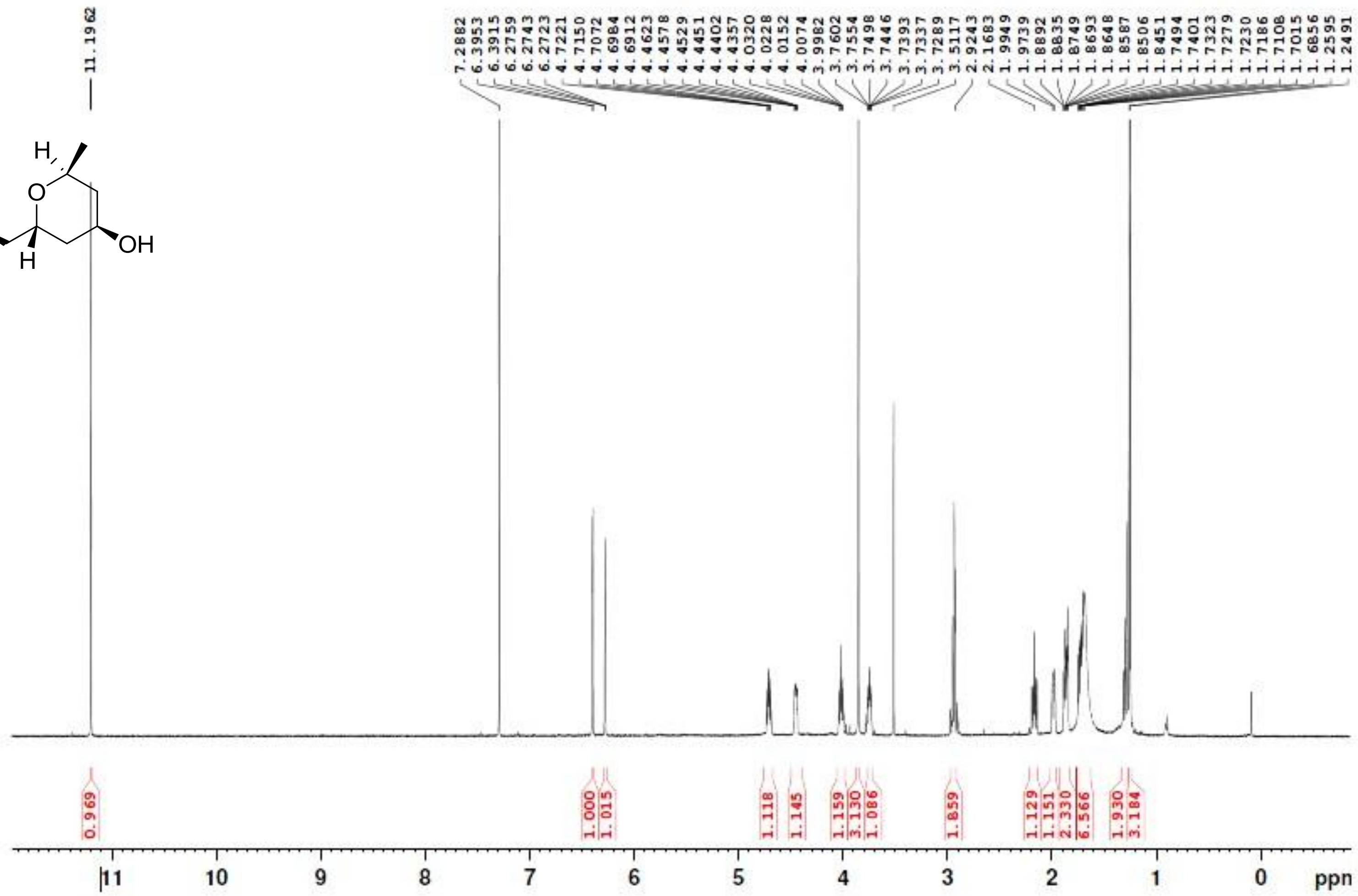

Figure S23. The C spectrum of compound **9**.

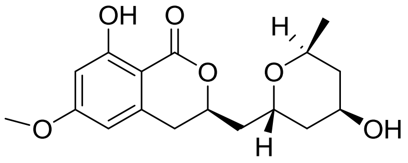

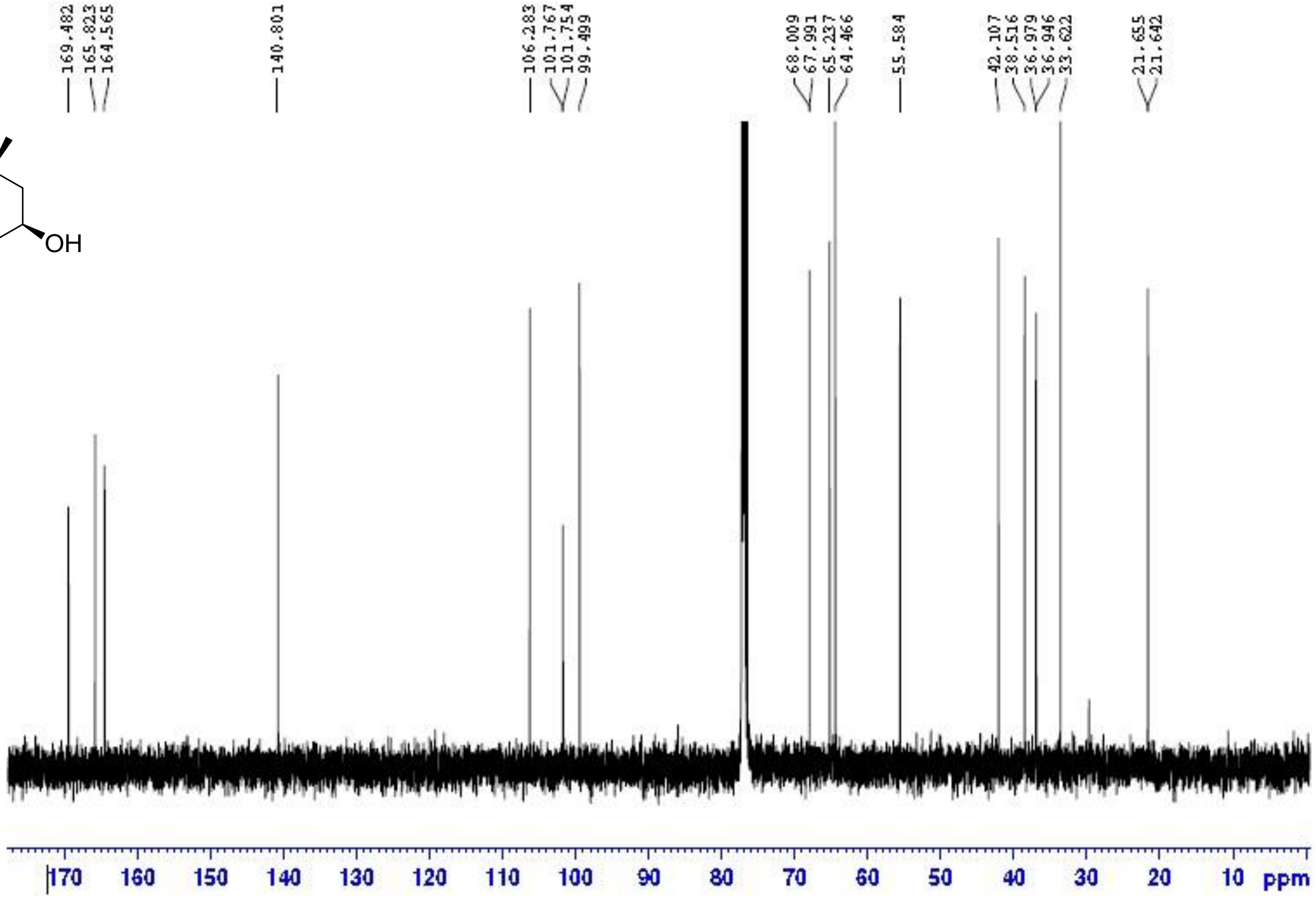

© 2014 by the authors; licensee MDPI, Basel, Switzerland. This article is an open access article distributed under the terms and conditions of the Creative Commons Attribution license (http://creativecommons.org/licenses/by/4.0/).
